# Nutritional Compositions, Phenolic Contents and Antioxidant Activities of Rainfed Rice Grown in Different Degrees of Soil Salinity

**DOI:** 10.3390/foods12152870

**Published:** 2023-07-28

**Authors:** Yuraporn Sahasakul, Amornrat Aursalung, Sirinapa Thangsiri, Piya Temviriyanukul, Woorawee Inthachat, Pirach Pongwichian, Kamontip Sasithorn, Uthaiwan Suttisansanee

**Affiliations:** 1 Food and Nutrition Academic and Research Cluster, Institute of Nutrition, Mahidol University, Salaya, Phuttamonthon, Nakhon Pathom 73170, Thailand; yuraporn.sah@mahidol.ac.th (Y.S.); amornrat.aur@mahidol.ac.th (A.A.); sirinapa.tha@mahidol.ac.th (S.T.); piya.tem@mahidol.ac.th (P.T.); woorawee.int@mahidol.ac.th (W.I.); 2 Land Development Department, Phaholyothin Rd., Lat Yao, Chatuchak, Bangkok 10900, Thailand; pirach3739@hotmail.com (P.P.); misskamonthip@gmail.com (K.S.)

**Keywords:** crop, fertilizer, minerals, nutrition, *Oryza sativa* L., rice agricultural, soil quality, vitamins

## Abstract

Rice (*Oryza sativa*) is a staple food crop for over half of the world’s population. However, drought as a result of climate change has led to increased soil salinity, thereby reducing agricultural potential, especially rice nutritional compositions and biochemical properties. Nevertheless, soil management by using suitable fertilizers might be able to improve rice quality even though these rice samples were grown in soil with a high degree of salinity. This study investigated nutritional compositions, phenolic contents, and antioxidant activities of twenty-five rainfed rice samples in Khao Dawk Mali 105 (KDML105) and Rice Department 15 (RD15) varieties grown in soil with different degrees of salinity. The soil, however, had been improved by the usage of fertilizer at the tillering and booting stages. Results indicated that all rice samples exhibited similar nutrients, total phenolic contents (TPCs), and antioxidant potentials, suggesting that appropriate fertilizer could improve rice qualities. Principle Component Analysis (PCA) and Pearson correlation results suggested that regardless of rice varieties, organic matter (OM) and soil potassium (Ks) showed a very strong positive correlation with protein and minerals (Ca, Na, K, and Fe), while opposite results were observed with soil pH. Moderate to very weak correlations were also observed between soil parameters and TPCs, as well as between soil parameters and antioxidant activities. The received information will be useful for the future development of appropriate fertilizer usage in salt-tolerant rice with particular nutritional quality.

## 1. Introduction

Rice (*Oryza sativa*) is one of the world’s most important food crops as the staple diet for over half of the global population [1], accounting for 20% of dietary energy intake [2]. Rice is widely consumed in its white or polished form [3]. White rice is a product of milled and dehusked rice, ridding the rice grain of its germ and rice bran. Brown rice, on the other hand, undergoes only the dehusking process, resulting in the removal of the husk inedible portion of rough rice or paddy rice [4]. Brown rice, with its bran and germ layer intact, is rich in fiber, minerals, vitamins, protein, and bioactive compounds, such as tocopherols, polyphenols, and γ-oryzanol, as a result of phytochemicals with antioxidant properties remaining in the bran and germ layer [5,6]. However, rice quality and quantity vary under disparate climate and soil conditions.

One of the main abiotic factors for rice growth is salinity [7]. Saline soil contains large amounts of soluble salts in solution that can be observed as patches of salt deposits, especially in the dry season. Salinity can be defined as the electrical conductivity (EC) of soil solutions extracted from water-saturated soils greater than 2 deci-Siemens/meter (dS/m) at 25 °C [8]. Salinity is a major global soil degradation problem, which occurs as a result of drought that reduces plant growth and production, leading to agricultural decline [9,10,11]. The impacts of salinity on rice growth and productivity, as well as nutrient accumulation in rice roots, shoots, and leaves, are well documented [9,10,11,12,13,14], but a few studies have addressed the nutritional compositions of rice grains [15,16,17]. Salt stress lowered amylose content in rice grains and increased total starch and protein contents [18]. Amounts of Na and Mg increased in rice grains under salt stress treatment, with highly salt-sensitive rice samples being the most influenced [13]. By contrast, salinity led to decreased K and Zn contents in rice grains, while Ca levels were unaffected [13]. Phenolics and health-related properties such as antioxidant potential were also impacted by salinity. Salt stress strongly impacted antioxidant potentials, especially antioxidant enzymes in rice leaves and shoots [10,19], but no impacts were reported in rice grains.

In Thailand, inland saline soils account for 1.904 million hectares, and soil salinity is a major problem in rice cultivation in the northeastern region [20]. Local farmers still cultivate rice in saline land; however, salinity causes rice grains to dehydrate, leading to nutrient imbalance and low productivity. One way to overcome low rice productivity resulting from soil salinity is to grow salinity-tolerant rice varieties. The well-recognized Khao Dawk Mali 105 (KDML105) and Rice Department 15 (RD15) rice varieties are moderately salt-tolerant but produce low grain yields in saline land. The Rice Department (Ministry of Agriculture and Cooperatives, Bangkok, Thailand) has improved several selected rice varieties with salt tolerance and high productivity as alternatives for farmers. The Land Development Department (Ministry of Agriculture and Cooperatives, Bangkok, Thailand) has also researched ways to increase rice productivity by emphasizing soil management with organic fertilizers together with planting salt-tolerant rice varieties. Previous research mainly concentrated on the effect of salinity on rice grown in growth-based materials or regular soils, or saline soils treated with different degrees of saline water in the laboratory or greenhouse [9,12,13,21]. However, scant research has been conducted on the quality of rice grown in saline soil treated with regular water (rain-fed rice) combined with appropriate fertilizer as out-of-laboratory or traditional rice agriculture for local farmers. Therefore, this research investigated the nutritional compositions, phenolic contents, and antioxidant potentials of twenty-five rice samples in moderately salinity-tolerant KDML105 and RD15 rice varieties grown in soil with different degrees of salinity and the usage of appropriate fertilizer. This information will provide valuable knowledge relating to the effect of soil improvement on the quality of rainfed rice grown in saline soil.

## 2. Materials and Methods

### 2.1. Sample Collection, Preparation, and Extraction

Twenty-five rice samples of two rice varieties, including KDML105 (18 samples) and RD15 (7 samples), and soil samples were collected from different locations, as indicated in Table 1, by the Land Development Department, Ministry of Agriculture and Cooperatives (Bangkok, Thailand) in February 2022. The usage of fertilizer was as follows: (i) tillering stage: 25 kg nitrogen (N)/ha and 7.8 kg phosphorus (P_2_O_5_)/ha; (ii) booting stage: 6.25 kg N/ha, 3.125 kg P_2_O_5_/ha and 26.56 kg potassium (K_2_O)/ha. There was no control (rice growth without fertilization) in this experiment because, without the usage of fertilization, rice product yield would be low. The characterizations of all rice samples were determined regarding their appearance and color using a ColorFlex EZ spectrophotometer (Hunter Associates Laboratory, Reston, VA, USA), as shown in Supplementary Table S1. All rice samples were then ground into a fine powder using a 600 W grinder (Philips Electronic Co., Ltd., Jakarta, Indonesia) and kept at −20 °C until analysis. Soil samples were collected using a field shovel by digging up to 30 cm deep, 15 cm × 15 cm wide (approximately 1 kg). The samples were kept in a plastic bag until analysis.

For rice extraction, rice (4 g) was extracted with 60% (*v*/*v*) aqueous ethanol (20 mL) at 50 °C for 2 h using a WNE45 water bath shaker (Memmert GmBh, Eagle, WI, USA) according to the previous report [22]. Centrifugation using a Hettich^®^ ROTINA 38R centrifuge (Andreas Hettich GmbH, Tuttlingen, Germany) at 3800× *g* for 15 min was performed to collect the supernatant before filtering through a 0.45 µM polytetrafluoroethylene (PTFE) syringe filter. The filtrate was kept at −20 °C until further analysis.

### 2.2. Determination of Soil Physicochemical Characteristics

#### 2.2.1. pH

The soil pH was determined using a glass electrode following the previous method [23]. Briefly, the soil sample (approximately 10 g) was mixed with distilled water (10 mL) in a ratio of 1:1. The mixture was stirred until completely dispersed and allowed to stand for 30 min. A Lab855 pre-calibrated pH meter (Sl Analytics GmbH, Mainz, Germany) was employed for the pH measurement of the soil suspension.

#### 2.2.2. Organic Matter

The organic matter (OM) was determined according to the Walkley–Black method [24]. Briefly, the soil sample (1 g) was mixed with 1 N potassium dichromate (10 mL) and concentrated sulfuric acid (15 mL) before swirling gently for 2 min and allowing it to stand for 30 min. To the mixture, distilled water (50 mL) was added, and the solution was allowed to cool to room temperature (25 °C) before adding *O*-phenanthroline (5 drops) as the indicator. The excess potassium dichromate in the mixture was titrated with 0.5 N ferrous ammonium sulfate solution until the mixture turned from greenish to red–brown. The volume of ferrous ammonium sulfate solution used was recorded. A blank was run in parallel by following the same steps without adding the soil sample. The organic carbon content was then calculated using Equation (1) as follows:

(1) $$\%\;\mathrm{Organic}\;\mathrm{carbon}=\;\frac{\left(B-T\right)N}{B}\;\times \;\frac{100}{77}\;\times \;3\;\times \;\frac{100}{10^{3}}\;\times \;\frac{10}{W},$$

where B is the volume of ferrous ammonium sulfate solution used to titrate with blank (mL); T is the volume of ferrous ammonium sulfate solution used to titrate with soil sample (mL); N is the concentration of potassium dichromate (N), and W is the weight of the soil sample (g). The result was then converted to OM using Equation (2) as follows:

(2) % Organic matter= % Organic carbon × 1.724,

where the factor of 1.724 accounted for the fact that organic carbon accounts for approximately 58% of soil OM.

#### 2.2.3. Available Potassium

The determination of available potassium (Ks) in soil was conducted using ammonium acetate as the extractant, as described previously [25]. Briefly, the soil sample (2.5 g) was dissolved in ammonium acetate solution (25 mL) and shaken in a New Brunswick Innova 2300, 51 mm shaker (Eppendorf, Hamburg, Germany) for 30 min before filtering through a Whatman No.5 filter paper (Whatman International Ltd., Kent, UK). The concentration of extracted potassium was measured by a model 420 flame photometer (Sherwood Scientific Ltd., Cambridge, UK) at the wavelength of 383 nm. A standard potassium solution at concentrations of 0, 5, 10, 15, and 20 mg/L was run parallel to calibrate and prepare a standard curve. The Ks were calculated using Equation (3) as follows:

(3) $$\mathrm{Available}\;\mathrm{potassium}\;(\mathrm{mg}/\mathrm{kg})=\frac{D\;\times \;\mathrm{df}\;\times \;B}{A},$$

where A is the weight of the soil sample (g); B is the volume of the ammonium acetate extract solution (mL); df is the dilution factor, and D is the concentration of potassium when compared with the standard set (mg/kg).

#### 2.2.4. Available Phosphorus

The determination of available phosphorus (Ps) in soil was determined following a method previously described [26]. Briefly, the soil sample (1 g) was dissolved in a Bray II solution containing 0.03 N ammonium fluoride and 0.1 N concentrated hydrochloric acid (10 mL). The mixture was shaken in a New Brunswick Innova 2300, 51 mm shaker (Eppendorf, Hamburg, Germany) for 1 min and filtered through a Whatman No. 5 filter paper (Whatman International Ltd., Kent, UK). The filtrate was then mixed with a sulfuric-molybdate-tartrate solution containing ascorbic acid in a ratio of 1:16. The mixture was left for 30 min, and the extracted Ps was monitored using a lambda 35 UV-vis spectrophotometer (Perkin Elmer, Waltham, MA, USA) at a wavelength of 882 nm. Blank and standard solutions (0, 2, 4, 6, 8, 10, and 15 mg/L) were run in parallel. The Ps were calculated using Equation (4) as follows:

(4) $$\mathrm{Available}\;\mathrm{phosphorus}\;(\mathrm{mg}/\mathrm{kg})=\frac{B\;\times \;\mathrm{df}\;\left(\mathrm{Sample}\right)\;\times \;R}{A\;\times \;\mathrm{df}\;(\mathrm{Standard})},$$

where A is the weight of the soil sample (g); B is the volume of the Bray II extract solution (mL); df is the dilution factor, and R is the readout value when compared with the standard set.

#### 2.2.5. Electrical Conductivity Extract

The electrical conductivity extract (EC_e_) was determined using a soil saturation extract based on a previous method [27]. First, the soil sample (approximately 400 g) was gradually dissolved in distilled water while stirring with a spatula until the soil paste became saturated. Soil saturation was characterized by a glistening appearance of soil paste as the light was reflected and a slight flow movement when the container was tipped and tilted. The soil mixture was left overnight and rechecked the next day to ensure saturation. Additional water would be added to the soil paste if the glistering characteristic were lost, while more soil would be added if the paste were excessively wet. Once the soil paste was fully saturated, a portion was taken to determine the moisture content of the saturated paste. Another portion of the saturated soil paste was transferred to the filter funnel with filter paper, and a vacuum was applied to collect the extract in a bottle. The EC_e_ was then measured using a Lab 955 conductivity meter (Sl Analytics GmbH, Mainz, Germany).

### 2.3. Analysis of Nutritional Compositions

The nutritional components of all rice samples were determined based on the standard procedures from the Association of Official Analytical Chemists (AOAC), 2019 [28]. The analysis complied with the ISO/IEC 17025:2017 international standard for laboratory quality systems at the Accredited Laboratory of the Institute of Nutrition, Mahidol University. The proximate compositions (moisture content, fat, protein, ash, total dietary fiber, total carbohydrate, and energy), minerals (calcium, sodium, potassium, magnesium, zinc, and iron), and vitamin B3 were analyzed, as previously reported with slight modifications [29].

The moisture content was evaluated by drying the samples in a hot-air oven (Memmert, Eagle, WI, USA) at 100 ± 5 °C until a constant weight was reached, following AOAC 931.04.

Total fat content was determined using an HT 1043 Tecator Soxtec System (Foss Tecator, Hoganas, Sweden) with acidic hydrolysis and solvent extraction based on AOAC 922.06.

Protein content was calculated using a conversion factor of 5.95 on total nitrogen determined using the Kjeldahl method based on AOAC 991.20.

Ash content was analyzed based on AOAC 930.30, which involved incinerating organic matters in a muffle furnace (Carbolite Gero Ltd., Hope, UK) at 550 ± 5 °C. The weight of the remaining residue was measured and reported as the ash content.

Total dietary fiber was analyzed according to the enzymatic–gravimetric method (AOAC 985.29).

Once the moisture, protein, fat, and ash contents were determined, they were subtracted from 100 to obtain the total carbohydrate content, as shown in Equation (5), while Atwater factors were used to calculate energy using Equation (6) as follows:

(5) Total carbohydrate=100 − (Moisture+Fat+Protein+Ash)

(6) Energy=(Protein × 4)+(Total carbohydrate × 4)+(Fat × 9)

Mineral levels, including calcium, sodium, potassium, magnesium, zinc, and iron in the ash residue, were determined using a flame atomic absorption spectrophotometer (AAS) (Thermo Electron Corporation, Cambridge, UK) in accordance with AOAC 985.35.

Vitamin B3 was determined by high-performance liquid chromatography (HPLC) analysis using an in-house method based on AOAC 961.14 [30,31].

### 2.4. Determination of Phenolic Profile and Total Phenolic Contents

Using rice extracts from Section 2.1, the solvent of selected rice extracts was evaporated using a DTC-22 diaphragm vacuum pump (EYELA CO., LTD., Shanghai, China) until dry. The dry extracts underwent acidic and non-acidic hydrolysis. For acidic hydrolysis, the dry extract (1 and 2 g) was re-dissolved in 62.5% (*v*/*v*) methanol containing 0.5 g *tert*-butylhydroquinone (tBHQ) (10 mL) and acidified with formic acid (40 mL). The reaction proceeded at 80 °C using a TW20 water bath shaker (Julabo GmbH, Seelbach, Germany) for 2 h before being put on ice for 5 min. Ascorbic acid (1% (*v*/*v*), 100 µL) was added to the mixture prior to sonication in an ultrasonic bath (Branson Ultrasonics Corp., Danbury, CT, USA) for 5 min. The final extract concentrations of 20 and 40 mg/mL were achieved by adjusting the volume with 62.5% (*v*/*v*) methanol containing 0.5 g tBHQ. For non-acidic hydrolysis, the dry extract was dissolved in 62.5% (*v*/*v*) methanol containing 0.5 g tBHQ to obtain the final 10 mg/mL concentration. Both extracts that underwent acidic and non-acidic hydrolysis were filtered through a 0.22 µM PTFE syringe filter for a liquid chromatography–electrospray ionization tandem mass spectrometry (LC-ESI-MS/MS) analysis, as previously reported [32].

Total phenolic contents (TPCs) of the rice extracts (from Section 2.1) were obtained using Folin–Ciocalteu phenol reagent and gallic acid standard (0–200 µg/mL), as previously reported [22] without any further modifications. The reaction at 765 nm was monitored utilizing a Synergy HT 96-well UV-visible microplate reader (BioTek Instruments, Inc., Winooski, VT, USA) with Gen 5 data analysis software (version 2.09). The results were presented as mg gallic acid equivalent (GAE)/g dry weight (DW).

### 2.5. Determination of Antioxidant Activities

Antioxidant activities of all rice extracts (from Section 2.1) were determined by ferric ion reducing antioxidant power (FRAP), 2,2-diphenyl-1-picrylhydrazyl (DPPH) radical scavenging and oxygen radical absorbance capacity (ORAC) assays based on a previous report [33] without any further modifications. Briefly, FRAP assay was examined utilizing 2,4,6-tri(2-pyridyl)-*S*-triazine and FeCl_3_·6H_2_O in acetate buffer as a reagent and end-point measurements at 600 nm as a detection wavelength. The other end-point DPPH radical scavenging assay used DPPH reagent in 95% (*v*/*v*) aqueous ethanol and a detection wavelength of 520 nm. The ORAC assay employed a kinetic reaction with 2,2′-azobis(2-amidinopropane) dihydrochloride and sodium fluorescein as reagents and an excitation wavelength of 485 nm and an emission wavelength of 528 nm as the detection wavelength. The reaction was monitored utilizing a microplate reader. Trolox was used as a standard, and the results were expressed as µmol Trolox equivalent (TE)/g DW.

### 2.6. Statistical Analysis

All experiments were carried out as three independent sets of samples (*n* = 3); each was performed in triplicate. The results were expressed as mean ± standard deviation (SD). Statistical analysis was performed through one-way analysis of variance (ANOVA) and Duncan’s multiple comparison tests with significant differences at *p* < 0.05 (a statistical package for the social sciences, version 18 for Windows, SPSS Inc., Chicago, IL, USA). The principal component analysis (PCA), agglomerative hierarchical clustering analysis (AHC), and Pearson correlation of soil parameters, nutritional compositions, TPCs, and antioxidant properties were analyzed using XLSTAT^®^ (Addinsoft Inc., New York, NY, USA).

## 3. Results

### 3.1. Soil Physicochemical Characteristics

Soil physicochemical characteristics, including pH, EC_e_, OM, Ks, and Ps, were investigated (Table 1). Soil pH of rice samples of the KDML105 variety ranged between 4.7 and 8.2, while a narrow range of 4.5–5.7 was found in rice samples of the RD15 variety. KDML105-B14 and RD15-58 rice samples were grown in locations with the highest pH, while KDML105-14 and RD15-42 rice samples were collected from areas with the lowest pH. The Soil Survey Manual 2017 (United States Department of Agriculture) (USDA) [8] defines soil with pH ranging between 4.5and 5.0 as very strongly acidic (including growth locations of KDML105-14, KDML105-49, KDML105-51, KDML105-53, KDML105-55, KDML105-57, RD15-42, RD15-44, and RD15-47 rice samples), soil with pH ranging between 5.1 and 5.5 as strongly acidic (including growth locations of KDML105-29, RD15-31, RD15-34, and RD15-40 rice samples), soil with pH ranging between 5.6 and 6.0 as moderately acidic (including growth location of RD15-58 rice sample), soil with pH ranging between 6.1 and 6.5 as slightly acid (including growth locations of KDML105-19, KDML105-T1, KDML105-T6, and KDML105-T2 rice samples), soil with pH ranging between 6.6 and 7.3 (including growth locations of KDML105-6, KDML105-T2, and KDML105-B18 rice samples) as neutral, soil with pH ranging between 7.4 and 7.8 as slightly alkaline (including growth location of KDML105-T4 rice sample), and soil with pH ranging between 7.9 and 8.4 as moderately alkaline (including growth locations of KDML105-9, KDML105-B11, and KDML105-B14 rice samples).

EC_e_ related to soil salinity ranged from 0.58 to 15.87 dS/m in the soil of the rice samples of the KDML105 variety and from 0.60 to 11.09 dS/m in the soil of the rice samples of the RD15 variety. Growth locations of KDML105-19 and RD15-42 rice samples recorded the highest EC_e_. The Soil Survey Manual 2017 (United States Department of Agriculture (USDA) [8] defines soil with EC_e_ less than 2 as non-saline (including growth locations of KDML105-6, KDML105-9, KDML105-14, KDML105-29, KDML105-49, KDML105-51, KDML105-53, KDML105-55, KDML105-57, KDML105-T1, KDML105-T2, KDML105-T4, KDML105-T6, KDML105-B11, KDML105-B18, RD15-31, RD15-34, RD15-40, and RD15-58 rice samples), soil with EC_e_ ranging between 2 and <4 as very slightly saline (including growth locations of KDML105-B2, KDML105-B14, and RD15-44 rice samples), soil with EC_e_ ranging between 4 and <8 as slightly saline (RD15-47), and soil with EC_e_ ranging between 8 and <16 as moderately saline (including growth locations of KDML105-19 and RD15-42 rice samples).

Percentages of OM in the soil of the rice samples of the KDML105 variety ranged from 0.25 to 2.49 and from 0.60 to 2.17 in the soil of the RD15 variety. The KDML105-55 and RD15-47 rice samples were collected from soil with the highest OM, while KDML105-B14 and RD15-44 rice samples were collected from soil with the lowest OM contents. Soil minerals, including Ks and Ps, ranged from 8 to 121 and from 2 to 36 mg/kg, respectively, in the soil of the rice samples of the KDML105 variety and 27–155 and 4–12 mg/kg, respectively, in the soil of the rice samples of the RD15 variety. The KDML105-49 and KDML105-19 rice samples were grown in soil with the highest Ks and Ps, respectively. Similarly, the RD15-47 and RD15-42 rice samples were collected from areas with the highest Ks and Ps, respectively.

Interestingly, soil extracts with high OM tended to exhibit high Ks, suggesting a possible correlation between these two parameters. EC_e_ was also possibly correlated with Ps, with high EC_e_ leading to high Ps. However, pH was not correlated with other soil properties. All rice varieties were further investigated for their nutritional compositions, TPCs, and antioxidant activities to assess how these nutritional and biochemical properties related to soil physicochemical characteristics. Rice samples of KDML105 and RD15 varieties with different EC_e_ values were selected for further analysis of phenolic profiles. The KDML105-19 and RD15-42 rice samples were selected as rice representatives grown in moderately saline soil, while KDML105-B2 and RD15-44 rice samples were selected as rice representatives grown in very slightly saline soil. Most rice samples were grown in non-saline soil, and four varieties, including KDML105-51, KDML105-29, RD15-34, and RD15-58 rice samples, were selected as representatives for further analysis in this group.

### 3.2. Nutritive Values

Nutritive values of all rice samples as per 100 g fresh weight (FW) were determined regarding proximate compositions (energy, protein, fat, carbohydrate, total dietary fiber (TDF), and ash), minerals (Ca, Na, K, Mg, Fe, and Zn), and vitamins (vitamin B3), as shown in Supplementary Tables S2 and S3. All nutritive values were calculated as per 100 g dry weight (DW) for accurate comparison among rice samples, as shown in Table 2, Table 3 and Table 4.

Even though statistical analysis suggested significantly different proximate values, all rice samples exhibited similar energy, protein, fat, carbohydrate, TDF, and ash contents (Table 2). The rice samples of the KDML105 variety exhibited 406.82–412.74 kcal of energy, 7.17–9.95 g of protein, 2.89–3.88 g of fat, 84.77–87.85 g of carbohydrate, 1.73–3.59 g of TDF, and 1.56–2.09 g of ash. Among the rice samples of the KDML105 variety, the KDML105-55 rice sample possessed the highest protein but the lowest fat content, resulting in being the lowest energy provider. On the other hand, the KDML105-51 rice sample with high fat and protein contents provided the highest energy. Similarly, the rice samples of the RD15 variety exhibited 407.92–410.15 kcal of energy, 7.66–10.11 g of protein, 2.87–3.38 g of fat, 84.89–87.69 g of carbohydrate, 2.83–4.34 g of TDF, and 1.70–1.47 g of ash. Among all rice samples of the RD15 variety, the RD15-34 rice sample with the lowest fat content provided the lowest energy, while the RD15-58 rice sample with relatively high protein, fat, and carbohydrate contents provided the highest energy.

As a trend, it was observed that soil pH (Table 2) seemed to have a negative correlation with protein content, in which rice samples grown in lower (acidic) soil pH tended to have greater protein contents than the one grown in higher soil pH. On the other hand, protein contents seemed to be high in the rice samples grown in soil with high OM and Ks, suggesting a possible correlation between these parameters. It was also observed that rice samples grown in soil with low pH tended to have lower carbohydrate content than the ones grown in high soil pH. Other relations between soil physicochemical characteristics and proximate compositions were unclear.

Among macrominerals (Ca, Na, K, and Mg), all rice samples (100 g DW) in both rice varieties predominantly contained K, followed by Mg, Na, and Ca, respectively (Table 3). All rice samples of the KDML105 variety exhibited 208.34–346.20 mg of K, 98.79–143.88 mg of Mg, 5.69–33.71 mg of Na, and 5.05–13.55 mg of Ca. Among these, the KDML105-19 rice sample exhibited the highest K, corresponding to its soil with a high EC_e_ value. Furthermore, the KDML105-57 rice sample exhibited the highest Mg and Ca contents, while the KDML105-55 rice sample exhibited the highest Na. In the rice samples of the RD15 variety, 211.39–281.41 mg of K, 95.06–128.98 mg of Mg, 12.01–36.48 mg of Na, and 9.13–13.36 mg of Ca were detected. The RD15-31 rice sample exhibited the highest K and Mg contents but the lowest Na and Ca contents. The RD15-34 rice sample gave the highest Ca content, and the RD15-42 rice sample had the highest Na content. High EC_e_ values in RD15-42 rice samples suggested that EC_e_ values were not correlated with a particular mineral detected in rice samples.

The two microminerals, Fe and Zn, were also investigated in all rice samples (Table 3). Results indicated that the rice samples of the KDML105 variety exhibited Fe ranging from 0.09 to 2.40 mg, while Zn ranged from 2.05 to 3.65 mg. Among all rice samples of the KDML105 variety, the KDML105-55 rice sample exhibited the highest Fe content, with the highest Zn content detected in the KDML105-14 rice sample. The rice samples of the RD15 variety also exhibited similar ranges of 0.79–1.52 mg Fe and 2.14–2.63 mg Zn. The RD15-58 rice sample exhibited the highest Fe content, while the RD15-31 rice sample exhibited the highest Zn content.

It was also observed that the rice samples grown in soil with high pH (Table 1) tended to contain lower amounts of Ca, Na, and Fe than those grown in soil with low pH. Conversely, opposite results were observed with OM and Ks, in which the rice samples grown in soil with high OM and Ks tended to contain greater Ca, Na, and Fe content than those grown in soil with low OM and Ks. Other relations between soil physicochemical characteristics and minerals remained unclear.

In the Thai Food Composition Database (Thai FCD), vitamin B3 was the only vitamin determined in all brown rice samples, with the highest amount among all detected vitamins [34]. All rice samples of the KDML105 variety exhibited 2.35–4.63 mg vitamin B3/100 g DW, while the ones in the RD15 variety exhibited vitamin B3 in a range of 2.68–4.89 mg/100 g DW (Table 4). Among the rice samples of the KDML105 variety, the KDML105-B18 rice sample exhibited the highest vitamin B3 content, while the KDML105-T2 rice sample showed the lowest. In the rice samples of the RD15 variety, the RD15-58 rice sample exhibited the highest B3 content, with the lowest content detected in the RD15-42 rice sample. No clear trend was observed regarding the relationship between soil physicochemical characteristics and vitamin B3 content.

### 3.3. Total Phenolic Contents and Phenolic Profile

The aqueous ethanolic extracts of all rice samples were analyzed for TPCs by a spectroscopic assay using Folinn–Ciocalteu phenol reagent (Table 4). Results indicated that the rice samples of the KDML105 variety exhibited TPCs ranging from 0.33 to 0.59 mg GAE/g DW, with the KDML105-B14 rice sample providing the highest (up to 1.8-fold higher than the others) and KDML105-6 rice sample the lowest TPCs. The rice samples of the RD15 variety exhibited a smaller range of TPCs ranging from 0.44 to 0.50 mg GAE/g DW, while the RD15-58 rice sample exhibited the highest TPC (1.1-fold higher than the others) and RD15-42 and RD15-47 rice samples the lowest. There was no clear trend in the relationship between soil physicochemical characteristics and TPCs.

The phenolic profiles of selected rice samples (including KDML105-19, KDML105-51, KDML105-B2, RD15-44, KDML105-29, RD15-42, RD15-34, and RD15-58 rice samples) were determined using LC-ESI-MS/MS. Different extraction methods, including acidic and non-acidic hydrolysis of selected rice samples, were performed before LC-ESI-MS/MS analysis. A wide range of extract concentrations (20–40 mg/mL extract for rice samples undergoing acidic hydrolysis and 10 mg/mL extract for samples undergoing non-acidic hydrolysis) were also investigated. Higher extract concentrations of rice samples undergoing acidic hydrolysis were employed in this experiment due to the possibility of acidic degradation of phenolics. However, no detected peak was observed using 24 phenolics including 3,4-dihydroxybenzoic acid, 4–hydroxybenzoic acid, (−)-epigallocatechin gallate, apigenin, cinnamic acid, chlorogenic acid, hesperidin, caffeic acid, *p*-coumaric acid, syringic acid, luteolin, genistein, kaempferol, myricetin, ferulic acid, quercetin, naringenin, sinapic acid, vanillic acid, rutin, rosmarinic acid, isorhamnetin, galangin, and gallic acid as the LC-ESI-MS/MS standards. Thus, modified extraction methods such as increasing extract concentration should be further investigated.

### 3.4. Antioxidant Potentials

Antioxidant potentials of aqueous ethanolic extracts of all rice samples were determined via DPPH radical scavenging, FRAP, and ORAC assays, as indicated in Table 4. All rice extracts exhibited a small range of DPPH radical scavenging activities (0.13–0.17 µmol TE/100 g DW). Among the rice samples of the KDML105 variety, KDML105-49 and KDML105-51 rice samples exhibited the highest DPPH radical scavenging activities (up to 1.3-fold higher than the others), while the lowest was found in the KDML105-B11 rice sample. Similar results were detected in the rice samples of the RD15 variety, with the RD15-47 rice sample exhibiting higher DPPH radical scavenging activities (up to 1.2-fold higher) than the others and the RD15-31 rice sample the lowest. Similar to DPPH radical scavenging activities, FRAP activities were also in a small range of 2.00–2.69 µmol TE/g DW. The KMD105-53 rice sample exhibited up to 1.3-fold higher FRAP activity than the others, while the KMD105-29 rice sample gave the lowest. In the rice samples of the RD15 variety, the RD15-40 rice sample exhibited the highest FRAP activity (up to 1.2-fold higher than the others), while the RD15-42 rice sample gave the lowest. By contrast, ORAC activities of the rice samples of the KMD105 variety were quite distinct from each other (9.65–19.50 µmol TE/g DW), with KDML105-49 rice sample exhibiting the highest ORAC activity (up to 2.0-fold higher than the others), and KDML105-B11 rice sample providing the lowest. Among the rice samples of the RD15 variety with ORAC activities of 13.41–17.33 µmol TE/g DW, the RD15-58 rice sample exhibited 1.3-fold higher ORAC activity than the rest, with the RD15-34 rice sample the lowest. No clear trend was observed in the relation between soil physicochemical characteristics and antioxidant activities.

### 3.5. Principal Component Analysis and Pearson Correlation

**Figure 1:**
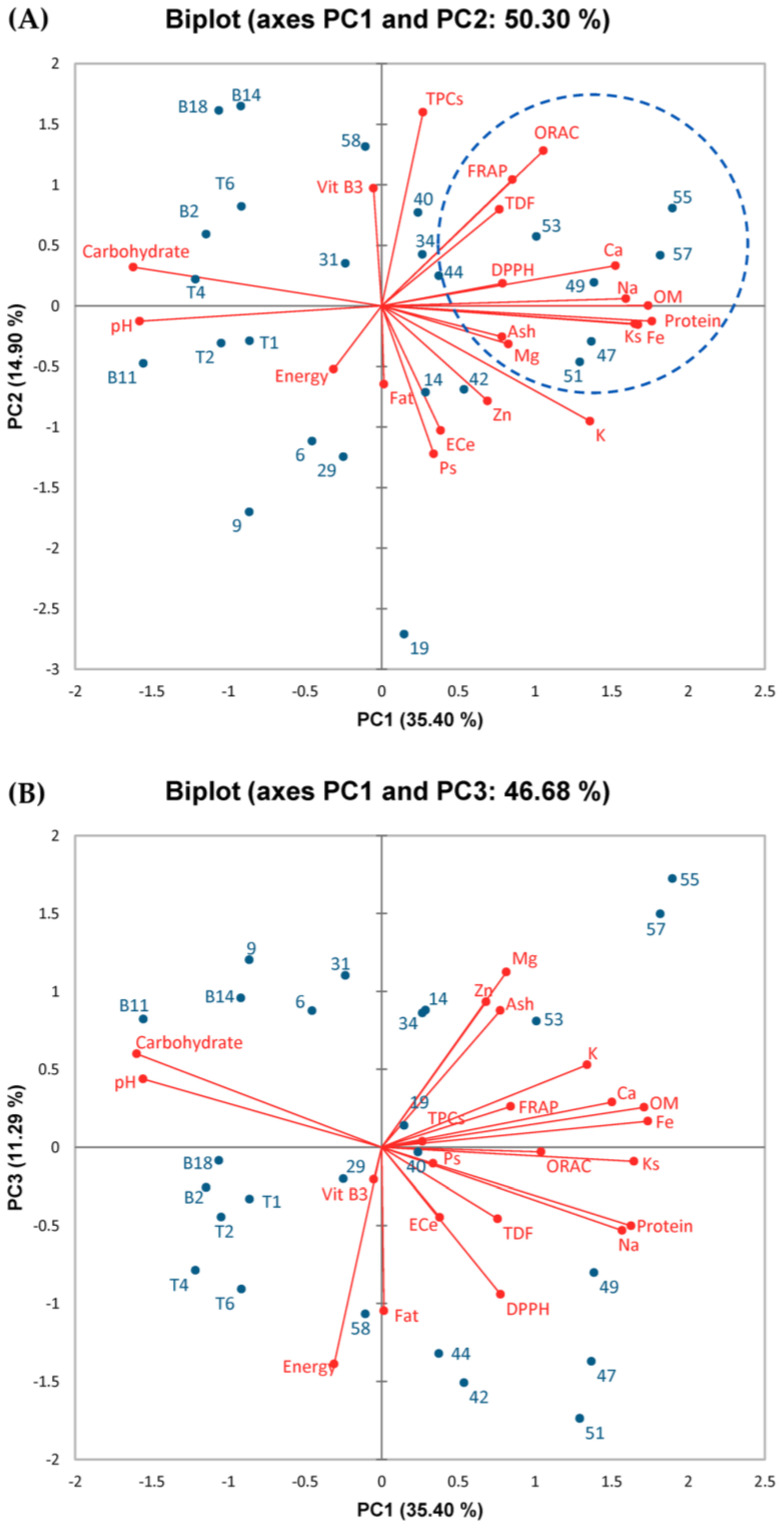
The biplot from principal component analysis (PCA) derived from the observations (25 rice samples) and mean values of all tested variables, including energy, protein, carbohydrate, fat, total dietary fiber (TDF), ash, vitamin B3 (Vit B3), Ca, Na, K, Mg, Fe, Zn, total phenolic contents (TPCs), antioxidant activities (DPPH radical scavenging, FRAP, and ORAC activities), and soil physicochemical characteristics (pH, organic matter (OM), soil potassium (Ks), soil phosphorus (Ps), and electrical conductivity extract (EC_e_)).

Copious information, including nutritive values, TPCs, antioxidant activities, and soil physicochemical characteristics (tested variables) of 25 brown rice varieties (observations), were examined, generating a complex analysis. To solve this issue, two independent statistical strategies, Principal Component Analysis (PCA) and the Pearson correlation coefficient, were performed to observe the correlation of all tested variables. PCA is a dimensionality reduction approach frequently used to decrease the dimensionality of big data while maintaining the greatest amount of information and interpretability, whereas the Pearson correlation coefficient assesses the linear connection between two variables. Mean values of the tested variables, including proximate compositions (energy, protein, fat, carbohydrate, TDF, and ash), mineral contents (Ca, Na, K, Mg, Fe, and Zn), vitamin B3 contents, TPCs, antioxidant activities (DPPH radical scavenging, FRAP, and ORAC activities), and soil physicochemical characteristics (pH, OM, Ks, Ps, and EC_e_) were subjected separately for PCA and Pearson correlation coefficient analysis, with results shown in Figure 1 and Table 5, respectively.

**Figure 2:**
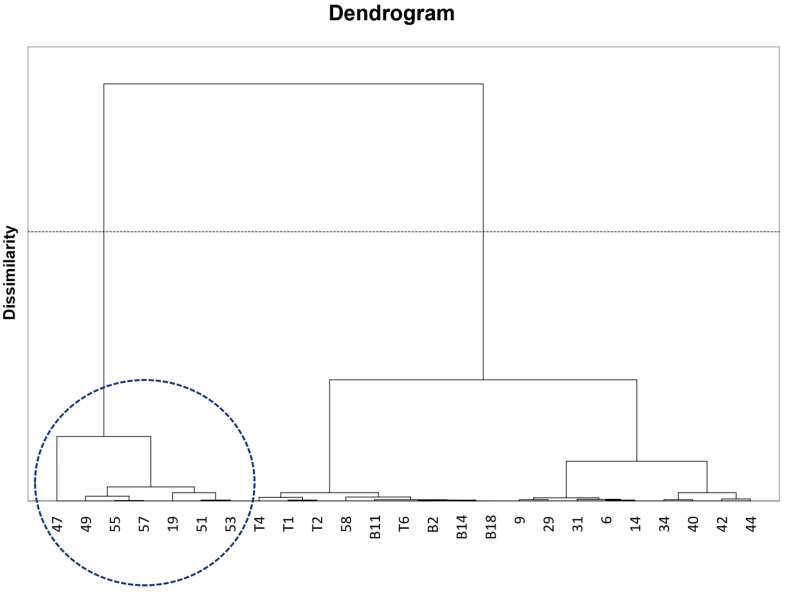
Dendrogram (similarity mode of agglomerative hierarchical clustering analysis) of 25 rice samples by mean value of all tested variables, including energy, protein, carbohydrate, fat, total dietary fiber (TDF), ash, vitamin B3 (Vit B3), Ca, Na, K, Mg, Fe, Zn, total phenolic contents (TPCs), antioxidant activities (DPPH radical scavenging, FRAP, and ORAC activities), and soil physicochemical characteristics (pH, organic matter (OM), soil potassium (Ks), soil phosphorus (Ps), and electrical conductivity extract (ECe)). The blue circle indicated the rice samples with high antioxidant activities, protein, and minerals.

For the PCA analysis, data were categorized into three PCs, PC1, PC2, and PC3. For all data, PC1 represented 35.40%; PC2 represented 14.90%, and PC3 represented 11.29%. Therefore, the PCA analysis explained 61.59% of all the tested variables. Protein, carbohydrate, Ca, Na, K, Fe, DPPH radical scavenging activities, pH, OM, and Ks were located in PC1, while TDF, vitamin B3, TPCs, FRAP activities, ORAC activities, Ps, and EC_e_ were positioned in PC2, and energy, fat, ash, Mg, and Zn were located in PC3 (Figure 1). In Figure 1, salinity, defined as the EC_e_ value, did not contribute to all investigated variables. The EC_e_ value depended on Ps but not on OM and Ks, indicating that elevated Ps may lead to increased EC_e_ or vice versa. Moreover, EC_e_ and Ps also had a positive effect on fat, Zn, and K. By contrast, a negative impact of EC_e_ was seen on TDF, vitamin B3, TPCs, as well as FRAP and ORAC activities, indicating that the rice samples grown under high EC_e_ conditions (such as KDML105-19, RD15-42, and RD15-47 rice samples) may have low TDF, vitamin B3, and TPCs attributable to poor antioxidant activities, especially FRAP and ORAC activities. However, other variables, including fat, Zn, and K, were not correlated with EC_e_ because PCA is a dimensionality reduction approach, which might lead to some errors. Furthermore, agglomerative hierarchical clustering analysis (AHC) was conducted in parallel. The mean data of all tested variables were used in the analysis, and Figure 2 shows that all 25 rice samples can be divided into two main groups. Interestingly, the group, which was indicated in the blue circle (Figure 2) consisted of seven rice samples covering RD15-47, KDML105-49, KDML105-55, KDML105-57, KDML105-19, KDML105-51, and KDML105-53. With the exception of KDML105-19, all six rice samples were consistent with the PCA data (blue circle in Figure 1), exhibiting high antioxidant activities, protein, and minerals. Overall, both PCA and AHC imply that RD15-47 might be the best rice variety among all tested varieties.

The matrix generated from the correlation coefficient (*r*) of soil parameters, nutritional compositions, phenolic contents, and antioxidant potentials of all rice samples is shown in Table 5. Meghanathan (2016) [35] considered that *r* positioned between ±0.8 and ±1 indicated a very strong correlation, between ±0.6 and ±0.79 a strong correlation, between ±0.4 and ±0.59 a moderate correlation, between ±0.2 and ±0.39 a weak correlation, and between ±0.0 and ±0.19 a very weak correlation. The strongest correlations were observed between parameters within the nutritional composition group. For example, energy was found to be strongly correlated with fat, suggesting that most energy was obtained from fat content rather than protein and carbohydrate. Among TPCs and antioxidant activities determined by DPPH radical scavenging, FRAP, and ORAC assays, only TPCs were found to be strongly correlated with ORAC activities. In the group of soil physicochemical characteristics, very strong correlations were observed between Ps and EC_e_ and between Ks and OM, while pH was negatively correlated with other soil parameters. These results corresponded to the PCA analysis. Between the different groups, soil physicochemical characteristics, including OM and Ks, formed strongly positive correlations with protein, Ca, Na, K, and Fe, while pH formed a strongly positive correlation with carbohydrates. In contrast, soil pH formed a strongly negative correlation with proteins, Ca, Na, and Fe. However, EC_e_ only formed weak and very weak correlations with nutritional compositions. Only moderate to very weak correlations were observed between soil physicochemical characteristics and the phenolic/antioxidant activity group.

A contradiction between PCA and Pearson correlation coefficients indicated inconclusive effects of EC_e_ on nutritive values, TPCs, and antioxidant properties. Thus, further research on how EC_e_ impacts those variables is recommended. Intriguingly, as indicated in Figure 1, OM and Ks were clearly associated with TPCs, antioxidant activities determined by FRAP and ORAC assays, and various nutritional parameters, including protein, TDF, Ca, Na, and Fe (within the blue circle), while PCA data were also supported by the Pearson correlation coefficient (*r* = 0.390 to 0.818) (Table 5). These results suggest that soil with high OM and Ks produces rice with high amounts of these mentioned variables.

## 4. Discussion

Soil salinity is a major problem regarding rice cultivation in Thailand, especially in the northeastern region of the country. Soil salinity affects the morphological properties of rice, including decreased plant height and root length, seeding growth, leaf growth, and mortality, as well as vegetative and productive phases [7]. Soil salinity also influences seedling biomass production, rice grain development, grain yield, harvesting index [7], and nutritional composition [15,16,17]. The Rice Department (Ministry of Agriculture and Cooperatives, Bangkok, Thailand) collected many salt-tolerant rice varieties, while the Land Development Department (Ministry of Agriculture and Cooperatives, Bangkok, Thailand) uses organic amendments and green manure to overcome the salinity problem. Many previous studies have reported on how soil salinity impacts rice quantity and quality, mostly emphasizing roots, shoots, and leaves, with a scant focus on rice grains. Most of these studies were performed in laboratory set-ups or greenhouses using growth-based material or regular soil treated with saline water. This study focused on rice grown in saline soil treated with regular water, imitating traditional rice agricultural management by local farmers (rainfed rice agriculture). The soil had been fertilized twice at the tillering and booting stages with a combination of nitrogen-phosphorus-potassium-containing fertilizers. Information on the nutritional compositions, phenolic contents, and antioxidant potentials of these salt-tolerant rice varieties can be applied in actual agriculture for further quality improvements in rice genetics. Results of the PCA and Pearson correlation suggested that (i) some soil parameters were strongly correlated with each other, for example, between OM and Ks and between EC_e_ and Ps, while (ii) some soil parameters were very strongly to strongly correlated with certain nutritional compositions. For example, OM and Ks were strongly correlated with proteins and some minerals, including Ca, Na, K, and Fe. In contrast, the opposite results were observed with soil pH, as it formed a strongly negative correlation with protein, Ca, Na, and Fe. Soil salinity, defined as EC_e_, was only weakly to very weakly correlated with nutritional compositions, TPCs, and antioxidant potentials, even showing a trend of negative correlation with TPCs and antioxidant activities determined by FRAP and ORAC assays.

### 4.1. Nutritional Compositions of Rice Grains

Our findings revealed carbohydrate to be the major constituent in both KDML105 and RD15 varieties of rice samples, with protein as the second most abundant component. Fat and fiber were present in similar amounts, whereas minerals and vitamins were found in lesser quantities, consistent with previous studies [36,37,38]. Nutritional analysis of KDML105 and RD15 rice varieties demonstrated that consuming 100 g of these rice types provided considerable amounts of vital nutrients, in accordance with Thai Recommended Daily Intakes (Thai RDIs) [39,40]. Specifically, the rice samples of the KDML105 variety provide up to 17, 26, 37, and 21% of Thai RDIs for carbohydrate, protein, Mg, and vitamin B3, respectively, while the ones in the RD15 variety supply up to 18, 26, 33, and 22% of these essential nutrients.

Rice has a relatively low protein content of 7–10% [41]; however, rice proteins have favorable amino acid profiles, including essential amino acids and sulfur-rich amino acids. Compared to other cereal proteins, rice proteins have higher levels of the essential amino acid lysine [42]. Rice proteins have well-balanced amino acid profiles, easy digestibility, hypoallergenic properties, and high nutritional quality [43,44]. Unlike wheat gluten, rice proteins contain glutelin, making rice flour a suitable ingredient for gluten-free products [43,45].

Supplementary Table S4 shows that Jasmine brown rice (KDML105) from the Thai Food Composition database (FCD, Food code A1) had similar levels of energy, protein, fat, carbohydrate, ash, and minerals to those found in our study [34]. However, dietary fiber content and vitamin B3 from the Thai FCD were 2.2 and 2.3 times higher than the minimum levels found in our rice samples of the KDML105 variety, respectively. Brown rice samples from unknown varieties in the Thai FCD (Food code A9) and the U.S. Department of Agriculture (USDA), Food Data Central (FDC, FDC ID 169703) had similar levels of proximate compositions and minerals to our study, while vitamin B3 from both databases was 2.7–3.1 times higher, with Zn content of the Thai FCD (Food code A9) 4.6–6.4 times lower than our rice samples [34,46]. These variations may be due to differences in rice varieties, genotypes, planting locations, environment, sample preparations, and analytical methods.

### 4.2. Correlations among Soil Physicochemical Characteristics

Soil physicochemical characteristics, including pH, EC_e_, OM, Ks, and Ps, were determined. Soil reaction or pH is defined as acidity or alkalinity due to hydrogen ions (H^+^) in the soil extract, with more H^+^ than OH^−^, giving acid soil pH. The electrical conductivity (EC) measures the soluble salts in the soil extract (or EC_e_) and is used to determine plant response to soil salinity. OM provides a significant source of nitrogen, phosphorus, and sulfur, which become available when plants and animals are decomposed by microorganisms. Ks and Ps measure the available K and available P, respectively, as the major minerals supporting plant growth.

Regular evaluation of soil chemical properties is crucial for effective land management to maximize crop yields [47]. We found that soil Ks content was highly correlated with OM content. Supportive evidence suggested that soil OM was positively correlated with soil Ps and Ks [48]. Most soil sampling sites were classified as non-saline, while some fell under the slightly to moderately saline categories. Elevated levels of soil EC_e_ indicated high levels of soil Ps, as demonstrated by EC_e_ values of 15.87 and 11.09, corresponding to Ps levels of 36 and 12, respectively. Evidence also suggested that soil EC and EC_e_ were correlated with certain forms of P and other minerals in the soil. For example, various fractions of phosphorus, including NaHCO_3_-Pi, NaOH-Pi, and NaOH-Po (inorganic-P: Pi and organic-P: Po fractions), were positively correlated with EC_e_, Ca, Mg, and Na in Egyptian soil [49]. By contrast, the NaHCO_3_-Po, HCl-P, and residual P fractions were negatively correlated with EC_e_, Na, and Cl [49]. In field-scale applications, assessing soil salinity by apparent soil electrical conductivity (EC_a_) is more practical. EC_e_ and EC_a_ are not identical, but they are highly related. EC_a_ indirectly reflects soil nutrient concentrations, with weak correlations observed between EC_a_ and OM, P, and K in soil from Indiana, USA [47], while moderate to very strong correlations between EC_a_ and P and moderate to strong correlations between EC_a_ and K were observed in soil samples from North Carolina, USA [50].

### 4.3. Effect of Soil Physicochemical Characteristics on Nutritional Compositions of Rice Grains

Salt stress causes physiological changes in the soil, which interfere with the establishment of healthy root systems in rice plants, leading to reduced nutrient uptake and lower grain yields [51,52]. Rice plants are highly susceptible to salinity stress, with a salinity threshold level of EC_e_ 3.0 dS/m. Exceeding this threshold causes a reduction in crop yield potential [53]. Previous research mainly focused on the effect of soil salinity on rice quality and quantity using rice grown in saline soil treated with saline water or regular soil treated with saline water. To the best of our knowledge, this is the first study to investigate the qualities of rainfed rice grown in soil with different EC_e_ values.

We found that EC_e_ was only weakly to very weakly correlated with rice nutritional compositions. This observation did not follow the same trend as previous investigations on rice grown in saline soil treated with saline water and regular soil treated with saline water. In these studies, salinity levels in soils had varying impacts on the nutritional compositions of rice grains. In low and moderate salinity soil (2 and 4 dS/m with these values kept constant for the whole experiment by treating with saline water), starch content in Nipponbare rice grains increased, while protein content remained unaffected [54]. However, increasing salinity in irrigation water (2, 4, and 6 dS/m) increased the grain protein content [55], while saline soil (EC 5–6 dS/m) resulted in increased protein and Na contents in three brown rice varieties from Pakistan [15]. The absorption and distribution of minerals in rice plants, including grains, are influenced by various factors such as soil composition, fertilization practices, climate change, and environmental stress [52]. Under saline water irrigation (25 mM NaCl), KDML105 rice grains showed decreased N, P, K, and Mg levels, while Na, Fe, Cu, and Zn levels increased [56]. Limited information is available regarding the impact of salinity on vitamin levels in rice grains, but studies on wheat indicated that environmental factors significantly affect the levels of B vitamins [57]. However, the effect of salinity on vitamin content of cereal grains still remains unclear.

Nevertheless, our results showed that OM and Ks were strongly correlated with protein and some minerals, including Ca, Na, K, and Fe, with no previous reports mentioning this finding. We also found that low soil pH also led to high protein, Ca, Na, and Fe in rice grains. A previous study also demonstrated higher Zn and Fe contents in rice grains grown in slightly acidic to neutral locations compared to those grown in alkaline locations [58]. A significant (*p* ≤ 0.001) and moderate negative correlation (*r* = −0.5) between soil pH and grain P content, as well as a significant (*p* ≤ 0.01) and weakly negative correlation (r = −0.3) between soil pH and grain Fe content were also found in basmati rice grain [59]. Although the correlation between soil pH and grain Zn content showed a similar tendency, it was not statistically significant [59].

## 5. Conclusions

Salinity is a major problem in rice agricultural management by retarding rice growth and productivity. Previous studies investigated the grain qualities of rice grown in growth-based materials treated with different degrees of saline water, but grain qualities of rice grown in saline soils treated with regular water or rainfed rice agriculture have not been assessed. We are the first to report on the nutritional compositions, TPCs, and antioxidant potentials of rainfed rice grown in soil under different degrees of salinity and the usage of particular fertilization. Our results indicated very strong correlations between soil parameters OM and Ks, as well as Ps and EC_e_. Interestingly, OM and Ks formed very strong to strong positive correlations with some nutrients, such as proteins and some minerals (Ca, Na, K, and Fe), while a strong negative correlation was observed between these nutrients and soil pH. Salinity is defined by EC_e_, but only weak and very weak correlations were observed between EC_e_ and nutritional compositions. Similar results were also observed between EC_e_ and TPCs, as well as between EC_e_ and antioxidant potentials. These observations suggested that soil salinity had little effect on nutritional compositions, TPCs, and antioxidant activities of rice under rainfed agricultural management. However, under particular fertilization, some soil physicochemical characteristics, including high OM and Ks and low pH, would potentially benefit some rice nutrients, such as protein, Ca, Na, and Fe. Nevertheless, increased sample sizes of rice grown in saline soil with high EC_e_ values should be further investigated to confirm our results.

## Figures and Tables

**Table 1:** Soil physicochemical characteristics of rice samples collected from different growth locations.

Rice Varieties	* Rice Code	Locations	Soil Quality				
			pH	OM (%)	Ks (mg/kg)	Ps (mg/kg)	EC_e_ (dS/m)
KDML105	14	15°27′33.7″ N 102°57′40.5″ E	4.7	1.52	41	5	1.03
	49	15°27′53.0″ N 102°58′04.8″ E	4.8	1.73	121	6	1.86
	55	15°27′52.9″ N 102°58′04.2″ E	4.8	2.49	99	5	1.37
	53	15°27′52.8″ N 102°58′04.3″ E	4.9	1.57	71	5	1.00
	51	15°27′52.7″ N 102°58′04.6″ E	5.0	1.90	74	4	1.53
	57	15°27′52.2″ N 102°58′04.0″ E	5.0	2.04	91	6	1.81
	29	15°27′43.0″ N 102°57′39.7″ E	5.5	0.96	25	5	0.58
	T6	15°03′52.4″ N 101°53′38.1″ E	6.1	0.46	11	3	0.83
	B2	15°03’53.4″ N 101°53′39.9″ E	6.4	0.71	17	4	2.07
	19	15°27′36.4″ N 102°57′38.5″ E	6.5	1.04	72	36	15.87
	T1	15°03′52.4″ N 101°53’38.1″ E	6.5	0.65	13	3	1.35
	B18	15°03′56.1″ N 101°53′31.7″ E	7.0	0.26	10	2	0.97
	6	15°27′27.9″ N 102°57′45.5″ E	7.0	0.46	35	3	1.31
	T2	15°03′52.4″ N 101°53′38.1″ E	7.3	0.62	13	3	1.09
	T4	15°03’52.4″ N 101°53′38.1″ E	7.4	0.38	8	3	0.93
	9	15°28′00.5″ N 102°57’42.4″ E	7.9	0.63	17	11	1.50
	B11	15°03′53.4″ N 101°53′39.9″ E	8.0	0.49	16	3	1.33
	B14	15°03′56.1″ N 101°53′31.7″ E	8.2	0.25	11	2	2.02
RD15	42	15°27′46.9″ N 102°57′58.8″ E	4.5	0.91	27	12	11.09
	44	15°27′46.8″ N 102°57′59.8″ E	4.7	0.60	49	11	3.53
	47	15°27′50.3″ N 102°58’06.2″ E	4.7	2.17	155	7	7.10
	34	15°27′42.6″ N 102°57′44.1″ E	5.1	1.72	45	4	1.51
	31	15°27’43.0″ N 102°57′44.9″ E	5.4	1.19	40	5	1.83
	40	15°27′56.1″ N 102°57′45.9″ E	5.4	1.37	52	6	0.61
	58	15°27′50.5″ N 102°57′52.4″ E	5.7	0.99	29	5	0.60

The Soil Survey Manual 2017 [8] defines soil with pH ranging between 4.5 and 5.0 as very strongly acidic (red), soil with pH ranging between 5.1 and 5.5 as strongly acidic (pink), soil with pH ranging between 5.6 and 6.0 as moderately acidic (orange), soil with pH ranging between 6.1 and 6.5 as slightly acidic (yellow), soil with pH ranging between 6.6 and 7.3 (grey) as neutral, soil with pH ranging between 7.4 and 7.8 as slightly alkaline (light blue), and soil with pH ranging between 7.9 and 8.4 as moderately alkaline (dark blue). In addition, soil with EC_e_ lower than 2 was defined as non-saline (light green), soil with EC_e_ ranging between 2 and <4 as very slightly saline (dark green), soil with EC_e_ ranging between 4 and <8 as slightly saline (light brown), and soil with EC_e_ ranging between 8 and <16 as moderately saline (dark brown). * Some rice samples, such as (i) T1, T2, T4, and T6, (ii) B2 and B11, and (iii) B14 and B18, were grown in the same location but in different trial planting plots. KDML105: Khao Dawk Mali 105; RD15: Rice Department 15; OM: organic matter; Ks: potassium in soil; Ps: phosphorus in soil; EC_e_: electrical conductivity extract; dS/m: deci-Siemens/meter.

**Table 2:** Proximate compositions including energy and contents of protein, fat, carbohydrate, total dietary fiber, and ash of 25 rice samples (per 100 g dry weight).

Rice Varieties	Rice Code	Nutritional Composition of Brown Rice (Per 100 g Dry Weight)					
		Energy (kcal)	Protein (g)	Fat (g)	Carb (g)	TDF (g)	Ash (g)
KDML105	14	409.19 ± 0.12 ^efgh^	8.64 ± 0.03 ^f^	3.15 ± 0.03 ^fgh^	86.56 ± 0.08 ^k^	2.84 ± 0.07 ^i^	1.64 ± 0.01 ^hi^
	49	410.46 ± 0.58 ^bcd^	9.63 ± 0.00 ^cd^	3.48 ± 0.12 ^bc^	85.16 ± 0.13 ^o^	3.31 ± 0.12 ^ef^	1.73 ± 0.00 ^d^
	55	406.82 ± 0.40 ^j^	9.95 ± 0.10 ^b^	2.89 ± 0.08 ^j^	85.24 ± 0.01 ^o^	3.32 ± 0.08 ^ef^	1.91 ± 0.01 ^b^
	53	408.51 ± 0.42 ^hi^	9.33 ± 0.05 ^e^	3.05 ± 0.06 ^ghij^	85.93 ± 0.07 ^m^	3.35 ± 0.04 ^def^	1.69 ± 0.03 ^efg^
	51	412.74 ± 0.05 ^a^	9.70 ± 0.05 ^c^	3.88 ± 0.00 ^a^	84.77 ± 0.05 ^q^	3.29 ± 0.09 ^ef^	1.66 ± 0.01 ^gh^
	57	407.89 ± 0.76 ^i^	9.59 ± 0.05 ^d^	3.25 ± 0.13 ^defg^	85.07 ± 0.07 ^op^	3.59 ± 0.02 ^bc^	2.09 ± 0.02 ^a^
	29	411.13 ± 0.37 ^b^	8.44 ± 0.04 ^g^	3.59 ± 0.05 ^b^	86.27 ± 0.01 ^l^	3.38 ± 0.02 ^de^	1.70 ± 0.03 ^def^
	T6	409.89 ± 1.10 ^cdef^	7.82 ± 0.03 ^kl^	3.29 ± 0.21 ^cdef^	87.25 ± 0.16 ^def^	2.90 ± 0.05 ^hi^	1.64 ± 0.02 ^kl^
	B2	410.23 ± 0.23 ^cde^	7.75 ± 0.02 ^lm^	3.29 ± 0.05 ^cdef^	87.41 ± 0.03 ^de^	2.83 ± 0.01 ^i^	1.56 ± 0.00 ^m^
	19	409.20 ± 0.35 ^efgh^	8.13 ± 0.08 ^h^	3.26 ± 0.09 ^def^	86.84 ± 0.19 ^ij^	2.84 ± 0.03 ^i^	1.77 ± 0.03 ^c^
	T1	410.79 ± 0.89 ^bc^	8.07 ± 0.01 ^h^	3.59 ± 0.16 ^b^	86.57 ± 0.16 ^k^	3.06 ± 0.01 ^g^	1.78 ± 0.02 ^c^
	B18	408.77 ± 0.71 ^ghi^	7.34 ± 0.00 ^o^	3.02 ± 0.16 ^hij^	88.04 ± 0.19 ^a^	3.31 ± 0.09 ^ef^	1.59 ± 0.03 ^lm^
	6	408.86 ± 0.04 ^ghi^	8.63 ± 0.01 ^f^	3.05 ± 0.01 ^ghij^	86.71 ± 0.02 ^jk^	1.73 ± 0.01 ^j^	1.60 ± 0.00 ^kl^
	T2	410.19 ± 0.19 ^cde^	8.05 ± 0.01 ^hi^	3.33 ± 0.00 ^cdef^	87.01 ± 0.06 ^ghi^	2.99 ± 0.01 ^gh^	1.61 ± 0.04 ^ijkl^
	T4	410.04 ± 0.18 ^cdef^	7.73 ± 0.01 ^mn^	3.43 ± 0.06 ^bcd^	87.06 ± 0.08 ^fghi^	3.22 ± 0.01 ^f^	1.78 ± 0.03 ^c^
	9	409.17 ± 0.25 ^efgh^	7.93 ± 0.04 ^j^	3.20 ± 0.06 ^efgh^	87.15 ± 0.03 ^fgh^	1.77 ± 0.02 ^j^	1.71 ± 0.01 ^de^
	B11	409.69 ± 0.61 ^defg^	7.17 ± 0.09 ^p^	3.29 ± 0.14 ^cdef^	87.85 ± 0.26 ^ab^	2.79 ± 0.05 ^i^	1.69 ± 0.03 ^efg^
	B14	407.97 ± 0.04 ^i^	7.97 ± 0.04 ^ij^	2.93 ± 0.03 ^fgh^	87.43 ± 0.01 ^d^	3.07 ± 0.04 ^g^	1.67 ± 0.02 ^fgh^
RD15	42	409.38 ± 0.50 ^efgh^	10.11 ± 0.09 ^a^	3.12 ± 0.10 ^fghi^	85.21 ± 0.18 ^o^	3.56 ± 0.10 ^bc^	1.56 ± 0.00 ^m^
	44	409.10 ± 0.18 ^fgh^	9.97 ± 0.01 ^b^	3.02 ± 0.02 ^hij^	85.50 ± 0.01 ^n^	3.56 ± 0.14 ^bc^	1.50 ± 0.02 ^n^
	47	410.06 ± 0.27 ^cdef^	10.03 ± 0.07 ^ab^	3.38 ± 0.06 ^cde^	84.89 ± 0.14 ^pq^	3.67 ± 0.02 ^b^	1.70 ± 0.01 ^def^
	34	407.92 ± 0.05 ^i^	8.05 ± 0.08 ^hi^	2.87 ± 0.02 ^j^	87.48 ± 0.04 ^cd^	3.47 ± 0.16 ^cd^	1.60 ± 0.01 ^jkl^
	31	408.47 ± 1.38 ^hi^	7.66 ± 0.06 ^n^	3.00 ± 0.29 ^hij^	87.69 ± 0.37 ^bc^	4.34 ± 0.00 ^a^	1.64 ± 0.02 ^hij^
	40	409.69 ± 0.13 ^defg^	7.90 ± 0.03 ^jk^	3.26 ± 0.04 ^def^	87.19 ± 0.02 ^efg^	2.83 ± 0.04 ^i^	1.65 ± 0.01 ^gh^
	58	410.15 ± 0.06 ^cde^	8.40 ± 0.02 ^g^	3.20 ± 0.02 ^efgh^	86.93 ± 0.05 ^hij^	3.51 ± 0.16 ^c^	1.47 ± 0.01 ^n^

All data were represented as mean ± standard deviation (SD) of triplicate experiments (*n* = 3). Lowercase letters indicate significantly different contents of the same proximate in different rice varieties at *p* < 0.05 using one-way analysis of variance (ANOVA) and Duncan’s multiple comparison test. KDML105: Khao Dawk Mali 105; RD15: Rice Department 15; Carb: carbohydrate; TDF: total dietary fiber.

**Table 3:** Mineral contents of 25 rice samples (per 100 g dry weight).

Rice Varieties	Rice Code	Mineral Contents of Brown Rice (mg/100 g Dry Weight)					
		Ca	Na	K	Mg	Fe	Zn
KDML105	14	8.19 ± 0.11 ^e^	20.83 ± 0.14 ^e^	293.23 ± 0.38 ^de^	124.28 ± 2.85 ^cde^	1.06 ± 0.06 ^ghi^	3.65 ± 0.26 ^a^
	49	9.48 ± 0.35 ^cd^	25.74 ± 0.49 ^d^	328.27 ± 5.89 ^bc^	126.60 ± 2.52 ^cde^	1.66 ± 0.08 ^c^	2.80 ± 0.07 ^cd^
	55	12.03 ± 0.27 ^b^	33.71 ± 1.85 ^ab^	326.62 ± 3.93 ^bc^	138.50 ± 0.14 ^ab^	2.40 ± 0.14 ^a^	2.88 ± 0.03 ^c^
	53	9.53 ± 0.41 ^cd^	23.71 ± 1.13 ^d^	322.82 ± 3.72 ^c^	131.03 ± 0.73 ^bc^	1.34 ± 0.02 ^def^	2.82 ± 0.02 ^c^
	51	9.80 ± 0.05 ^cd^	31.14 ± 4.65 ^bc^	334.55 ± 10.30 ^b^	129.09 ± 3.05 ^cd^	1.63 ± 0.01 ^c^	2.76 ± 0.07 ^cde^
	57	13.55 ± 1.35 ^a^	26.02 ± 2.03 ^d^	326.99 ± 3.05 ^bc^	143.88 ± 1.70 ^a^	2.06 ± 0.34 ^b^	2.90 ± 0.01 ^c^
	29	7.68 ± 0.41 ^ef^	11.34 ± 1.57 ^gh^	297.46 ± 6.64 ^de^	130.54 ± 1.54 ^bc^	0.96 ± 0.05 ^ij^	2.92 ± 0.03 ^c^
	T6	6.27 ± 1.03 ^ij^	10.14 ± 1.14 ^h^	210.46 ± 15.82 ^jk^	110.02 ± 18.64 ^fg^	0.49 ± 0.04 ^l^	2.11 ± 0.08 ^j^
	B2	5.77 ± 0.08 ^jk^	6.31 ± 0.60 ^i^	211.19 ± 4.51 ^jk^	109.68 ± 1.48 ^ef^	0.12 ± 0.04 ^mn^	2.51 ± 0.05 ^fgh^
	19	7.86 ± 0.27 ^ef^	19.43 ± 2.26 ^e^	346.20 ± 4.72 ^a^	131.44 ± 0.94 ^bc^	1.19 ± 0.22 ^fgh^	2.58 ± 0.08 ^efg^
	T1	5.74 ± 0.01 ^kj^	13.09 ± 2.65 ^fg^	237.24 ± 1.26 ^i^	131.27 ± 1.97 ^bc^	0.18 ± 0.02 ^mn^	2.07 ± 0.04 ^j^
	B18	7.22 ± 0.23 ^f^	11.53 ± 0.24 ^gh^	208.34 ± 2.94 ^jk^	117.15 ± 1.49 ^ef^	0.09 ± 0.01 ^n^	2.23 ± 0.06 ^ij^
	6	9.59 ± 0.04 ^cd^	14.44 ± 1.73 ^fg^	300.69 ± 3.29 ^d^	120.13 ± 0.43 ^de^	0.99 ± 0.01 ^hij^	2.89 ± 0.01 ^c^
	T2	6.06 ± 0.17 ^j^	11.95 ± 0.32 ^fgh^	234.16 ± 5.04 ^jk^	118.59 ± 3.82 ^ef^	0.34 ± 0.06 ^lm^	2.08 ± 0.03 ^j^
	T4	5.05 ± 0.48 ^k^	14.84 ± 0.27 ^f^	235.34 ± 3.12 ^i^	98.79 ± 0.85 ^h^	0.32 ± 0.00 ^lm^	2.05 ± 0.01 ^j^
	9	7.02 ± 0.03 ^fi^	14.20 ± 2.32 ^fg^	289.91 ± 1.23 ^ef^	119.46 ± 1.94 ^e^	0.73 ± 0.10 ^k^	3.30 ± 0.39 ^b^
	B11	6.16 ± 0.24 ^j^	5.69 ± 0.47 ^i^	219.90 ± 8.91 ^j^	122.59 ± 7.68 ^cde^	0.46 ± 0.21 ^l^	2.57 ± 0.10 ^efg^
	B14	7.69 ± 0.11 ^ef^	10.93 ± 1.58 ^h^	215.86 ± 4.28 ^jk^	117.08 ± 7.90 ^ef^	0.32 ± 0.02 ^lm^	2.57 ± 0.00 ^efg^
RD15	42	10.37 ± 0.52 ^c^	36.48 ± 3.24 ^a^	246.79 ± 3.15 ^h^	96.77 ± 1.64 ^h^	1.45 ± 0.23 ^cde^	2.45 ± 0.03 ^fgh^
	44	9.39 ± 0.80 ^d^	35.40 ± 1.50 ^a^	249.14 ± 7.10 ^h^	95.06 ± 6.95 ^h^	1.25 ± 0.15 ^efg^	2.41 ± 0.07 ^gh^
	47	11.83 ± 0.16 ^b^	34.34 ± 0.85 ^a^	259.06 ± 0.79 ^g^	119.26 ± 1.92 ^e^	1.22 ± 0.16 ^fg^	2.50 ± 0.01 ^fgh^
	34	13.36 ± 0.66 ^a^	29.90 ± 0.06 ^c^	247.55 ± 0.04 ^h^	124.63 ± 0.84 ^cde^	1.28 ± 0.08 ^efg^	2.33 ± 0.03 ^hi^
	31	9.13 ± 0.35 ^d^	12.01 ± 0.24 ^fgh^	281.41 ± 1.04 ^f^	128.98 ± 1.24 ^cd^	0.79 ± 0.13 ^jk^	2.63 ± 0.00 ^def^
	40	12.93 ± 0.83 ^a^	26.45 ± 1.19 ^d^	263.11 ± 5.01 ^g^	123.23 ± 1.01 ^cde^	1.05 ± 0.01 ^ghi^	2.14 ± 0.00 ^j^
	58	10.38 ± 0.29 ^c^	26.02 ± 0.02 ^d^	211.36 ± 4.64 ^jk^	107.58 ± 4.46 ^g^	1.52 ± 0.07 ^cd^	2.17 ± 0.04 ^ij^

All data were represented as mean ± standard deviation (SD) of triplicate experiments (*n* = 3). Lowercase letters indicate significantly different content of the same mineral in different rice varieties at *p* < 0.05 using one-way analysis of variance (ANOVA) and Duncan’s multiple comparison test. KDML105: Khao Dawk Mali 105; RD15: Rice Department 15.

**Table 4:** Vitamin B3, total phenolic contents (TPCs), and antioxidant activities of 25 rice samples.

Rice Varieties	Rice Code	Vitamin B3 (mg/100 g DW)	TPCs (mg GAE/g DW)	Antioxidant Activities		
				DPPH Radical Scavenging Activity (µmol TE/100 g DW)	FRAP Activity (µmol TE/g DW)	ORAC Activity (µmol TE/g DW)
KDML105	14	2.91 ± 0.00 ^l^	0.41 ± 0.02 ^jkl^	0.14 ± 0.01 f^ghi^	2.14 ± 0.10 ^fg^	16.95 ± 0.92 ^bc^
	49	4.28 ± 0.02 ^ef^	0.49 ± 0.02 ^def^	0.17 ± 0.01 ^ab^	2.34 ± 0.09 ^cd^	19.50 ± 1.46 ^a^
	55	4.17 ± 0.03 ^fg^	0.56 ± 0.04 ^ab^	0.15 ± 0.01 ^fghi^	2.31 ± 0.19 ^de^	19.09 ± 1.69 ^a^
	53	2.68 ± 0.01 ^m^	0.52 ± 0.04 ^cd^	0.15 ± 0.01 ^cdef^	2.69 ± 0.16 ^a^	17.16 ± 1.46 ^b^
	51	2.99 ± 0.07 ^kl^	0.48 ± 0.02 ^efg^	0.17 ± 0.01 ^ab^	2.53 ± 0.12 ^b^	15.74 ± 1.06 ^bcde^
	57	4.06 ± 0.00 ^g^	0.52 ± 0.02 ^cd^	0.15 ± 0.01 ^efgh^	2.46 ± 0.14 ^b^	18.07 ± 1.76 ^b^
	29	3.30 ± 0.01 ^i^	0.38 ± 0.02 ^l^	0.14 ± 0.01 ^fghi^	2.00 ± 0.15 ^i^	13.47 ± 1.09 ^fgh^
	T6	4.39 ± 0.00 ^de^	0.49 ± 0.03 ^def^	0.16 ± 0.02 ^abc^	2.19 ± 0.09 ^efg^	16.26 ± 0.79 ^bcde^
	B2	3.79 ± 0.04 ^h^	0.54 ± 0.03 ^bc^	0.14 ± 0.01 ^hij^	2.26 ± 0.06 ^def^	15.50 ± 1.54 ^cde^
	19	3.81 ± 0.04 ^h^	0.41 ± 0.02 ^lm^	0.15 ± 0.01 ^cdef^	2.08 ± 0.14 ^hi^	10.15 ± 0.95 ^i^
	T1	3.17 ± 0.03 ^ij^	0.47 ± 0.04 ^fghi^	0.14 ± 0.01 ^cdef^	2.16 ± 0.04 ^fg^	13.76 ± 1.06 ^fg^
	B18	4.63 ± 0.02 ^b^	0.55 ± 0.04 ^bc^	0.16 ± 0.01 ^abcd^	2.29 ± 0.12 ^de^	17.04 ± 1.54 ^bc^
	6	2.97 ± 0.01 ^kl^	0.33 ± 0.01 ^m^	0.15 ± 0.01 ^defg^	2.25 ± 0.13 ^def^	13.31 ± 1.18 ^gh^
	T2	2.35 ± 0.04 ^n^	0.44 ± 0.02 ^hi^	0.15 ± 0.01 ^fghi^	2.25 ± 0.11 ^def^	12.01 ± 1.17 ^h^
	T4	4.35 ± 0.02 ^de^	0.47 ± 0.03 ^fghi^	0.15 ± 0.01 ^fghi^	2.08 ± 0.07 ^hi^	13.57 ± 1.16 ^fgh^
	9	3.09 ± 0.04 ^jk^	0.36 ± 0.02 ^kl^	0.14 ± 0.01 ^fghi^	2.11 ± 0.11 ^hi^	12.01 ± 0.98 ^h^
	B11	4.03 ± 0.02 ^fg^	0.45 ± 0.05 ^ghi^	0.13 ± 0.00 ^fg^	2.08 ± 0.12 ^hi^	9.65 ± 0.81 ^i^
	B14	4.62 ± 0.06 ^c^	0.59 ± 0.06 ^a^	0.14 ± 0.02 ^fghi^	2.43 ± 0.14 ^bc^	17.34 ± 1.67 ^b^
RD15	42	2.68 ± 0.05 ^m^	0.44 ± 0.01 ^ij^	0.15 ± 0.00 ^efgh^	2.09 ± 0.05 ^hi^	15.33 ± 0.97 ^de^
	44	3.68 ± 0.04 ^h^	0.47 ± 0.02 ^fghi^	0.15 ± 0.01 ^efgh^	2.28 ± 0.14 ^de^	14.48 ± 1.01 ^gh^
	47	4.46 ± 0.04 ^cd^	0.44 ± 0.02 ^ijk^	0.16 ± 0.01 ^bcde^	2.16 ± 0.15 ^fg^	15.03 ± 1.31 ^ef^
	34	2.85 ± 0.13 ^lm^	0.45 ± 0.03 ^ghi^	0.14 ± 0.01 ^fghi^	2.29 ± 0.09 ^de^	13.41 ± 1.12 ^gh^
	31	3.27 ± 0.06 ^i^	0.47 ± 0.04 ^fghi^	0.13 ± 0.01 ^ij^	2.29 ± 0.09 ^de^	13.84 ± 1.20 ^fg^
	40	4.12 ± 0.33 ^fg^	0.47 ± 0.02 ^efgh^	0.15 ± 0.01 ^defg^	2.47 ± 0.11 ^b^	16.67 ± 1.53 ^bcd^
	58	4.89 ± 0.02 ^a^	0.50 ± 0.02 ^de^	0.15 ± 0.01 ^efgh^	2.30 ± 0.08 ^de^	17.33 ± 1.69 ^bc^

All data were represented as mean ± standard deviation (SD) of triplicate experiments (*n* = 3). Lowercase letters indicate significantly different values in different rice samples at *p* < 0.05 using one-way analysis of variance (ANOVA) and Duncan’s multiple comparison test. KDML105: Khao Dawk Mali 105; RD15: Rice Department 15; GAE: gallic acid equivalent; DW: dry weight; DPPH: 2,2-diphenyl-1-picrylhydrazyl; FRAP: ferric ion reducing antioxidant power; ORAC: oxygen radical absorbance capacity; TE: Trolox equivalent.

**Table 5:** Correlation matrix using correlation coefficient (*r*) of soil physicochemical characteristics, nutritional compositions, total phenolic contents, and antioxidant activities of rice.

	Energy	Protein	Fat	Carb	TDF	Ash	Ca	Na	K	Mg	Fe	Zn	B3	TPCs	DPPH	FRAP	ORAC	pH	OM	Ks	Ps	EC_e_
**Energy**	**1**																					
**Protein**	−0.022	**1**											**Correlation coefficient and its strength**									
**Fat**	**0.910**	0.064	**1**										**Very strong**		0.80 < *r* < 1.00 and −0.80 < *r* < −1.00							
**Carb**	−0.161	**−0.960**	−0.313	**1**									**Strong**		0.60 < *r* < 0.79 and −0.60 < *r* < −0.79							
**TDF**	−0.054	0.309	−0.040	−0.280	**1**								**Moderate**		0.40 < *r* < 0.59 and −0.40 < *r* < −0.59							
**Ash**	−0.268	0.200	0.154	−0.346	0.039	**1**							**Weak**		0.20 < *r* < 0.39 and −0.20 < *r* < −0.39							
**Ca**	−0.373	**0.574**	−0.295	**−0.484**	0.335	0.204	**1**						**Very weak**		0.00 < *r* < 0.19 and −0.00 < *r* < −0.19							
**Na**	−0.116	**0.838**	−0.096	**−0.755**	0.387	0.054	**0.774**	**1**														
**K**	−0.066	**0.522**	0.161	**−0.586**	−0.028	**0.533**	**0.406**	0.382	**1**													
**Mg**	−0.176	0.065	0.110	−0.171	0.031	**0.676**	0.323	−0.027	**0.654**	**1**			**Nutritional compositions**									
**Fe**	−0.232	**0.784**	−0.083	**−0.746**	0.287	0.359	**0.795**	**0.802**	**0.690**	0.370	**1**											
**Zn**	−0.190	0.298	−0.080	−0.289	−0.270	0.266	0.154	0.078	**0.635**	**0.420**	0.386	**1**										
**B3**	−0.101	−0.129	−0.049	0.114	0.164	0.128	0.033	−0.047	−0.306	−0.138	−0.018	−0.299	**1**									
**TPCs**	−0.254	0.040	−0.208	−0.004	**0.473**	0.123	0.085	0.062	−0.263	0.024	0.035	−0.320	**0.511**	**1**								
**DPPH**	0.308	0.394	0.314	**−0.436**	0.034	−0.003	0.192	0.359	0.260	0.002	0.264	−0.105	0.190	0.125	**1**		**Phenolic contents and antioxidant potentials**					
**FRAP**	−0.172	0.268	−0.143	−0.223	0.203	0.078	**0.453**	0.288	0.246	0.296	0.331	0.001	0.007	**0.561**	0.347	**1**						
**ORAC**	−0.196	**0.454**	−0.137	**−0.404**	0.322	0.148	**0.429**	0.393	0.121	0.114	**0.441**	0.123	0.368	**0.640**	**0.427**	**0.581**	**1**					
**pH**	0.007	**−0.742**	−0.012	**0.691**	**−0.570**	−0.045	**−0.674**	**−0.777**	**−0.436**	−0.156	**−0.726**	−0.199	0.149	−0.073	−0.299	−0.278	**−0.506**	**1**				
**OM**	−0.136	**0.648**	0.056	**−0.666**	0.390	**0.456**	**0.752**	**0.685**	**0.643**	**0.580**	**0.818**	0.364	−0.053	0.131	0.241	0.372	**0.444**	**−0.752**	**1**	**Soil parameters**		
**Ks**	−0.077	**0.718**	0.095	**−0.733**	0.316	**0.404**	**0.650**	**0.664**	**0.626**	**0.427**	**0.722**	0.282	0.153	0.068	**0.432**	0.300	0.372	**−0.638**	**0.846**	**1**		
**Ps**	−0.065	0.134	−0.023	−0.131	−0.079	0.102	0.052	0.216	**0.417**	0.051	0.228	0.111	−0.046	−0.306	0.082	−0.268	−0.366	−0.080	0.063	0.232	**1**	
**EC_e_**	−0.043	0.273	−0.035	−0.245	0.086	0.022	0.088	0.309	0.231	−0.110	0.178	−0.027	−0.048	−0.215	0.092	−0.324	−0.311	−0.147	0.068	0.278	**0.866**	**1**

Values in bold are different from 0 with a significance level α = 0.05; Carb: carbohydrate; TDF: total dietary fiber; B3: vitamin B3; TPCs: total phenolic contents; DPPH: 2,2-diphenyl-1-picrylhydrazyl; FRAP: ferric ion reducing antioxidant power; ORAC: oxygen radical absorbance capacity; OM: organic matter; Ks: potassium in soil; Ps: phosphorus in soil; EC_e_: electrical conductivity extract.

## Data Availability

The data used to support the findings of this study can be made available by the corresponding author upon request.

## Supplementary Materials

The following supporting information can be downloaded at: https://www.mdpi.com/article/10.3390/foods12152870/s1, Table S1: Appearance and color of twenty-five brown rice samples used in this experiment; Table S2: Proximate compositions, including energy and contents of protein, fat, carbohydrate, total dietary fiber, and ash, of brown rice samples (per 100 g fresh weight); Table S3: Mineral and vitamin B3 contents of brown rice samples (per 100 g fresh weight); Table S4: Proximate compositions of brown rice samples (KDML105 and RD15 varieties) compared with data from the Thai Food Composition Database (FCD) and the U.S. Department of Agriculture (USDA) FDC Databases (per 100 g dry weight).
