# Supplementary material for: Nutritional Compositions, Phenolic Contents and Antioxidant Activities of Rainfed Rice Grown in Different Degrees of Soil Salinity
Source: Foods. 2023 Jul 28;12(15):2870. doi: 10.3390/foods12152870 (PMC10417330; doi:10.3390/foods12152870)
Supplement: Supplementary file 1 [file foods-12-02870-s001.zip › foods-2507096-supplementary.pdf]

Supplementary materials

Nutritional Compositions, Phenolic Contents and Antioxidant Activities of Rainfed Rice Grown in Different Degrees of Soil Salinity

Yuraporn Sahasakul [1], Amornrat Aursalung [1], Sirinapa Thangsiri [1], Piya Temviriyanukul [1], Woorawee Inthachat [1], Pirach Pongwichian [2], Kamontip Sasithorn [2] and Uthaiwan Suttisansanee [1,*]

[1] Food and Nutrition Academic and Research Cluster, Institute of Nutrition, Mahidol University, Salaya, Phuttamonthon, Nakhon Pathom 73170, Thailand; yuraporn.sah@mahidol.ac.th (Y.S.); amornrat.aur@mahidol.ac.th (A.A.); sirinapa.tha@mahidol.ac.th (S.T.); piya.tem@mahidol.ac.th (P.T.); woorawee.int@mahidol.ac.th (W.I.)

[2] Land Development Department, Phaholyothin Rd., Lat Yao, Chatuchak, Bangkok 10900, Thailand; pirach3739@hotmail.com (P.P.); misskamonthip@gmail.com (K.S.)

* Correspondence: uthaiwan.sut@mahidol.ac.th; Tel.: +66-(0)-2800-2380 (ext. 422)

Supplementary Table S1:

Table S1. Appearance and color of twenty-five brown rice samples used in this experiment.

Rice Varieties	Rice No.	Appearance	Color Analysis		
			L*	a*	b*
KDML105	6	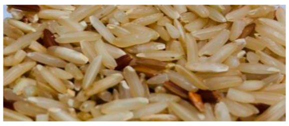	60.03 ± 0.56	5.78 ± 0.09	23.99 ± 0.10
	9	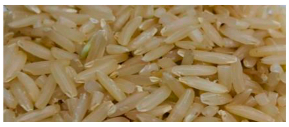	65.19 ± 0.05	4.95 ± 0.02	24.67 ± 0.02
	14	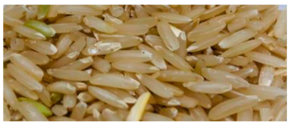	62.69 ± 0.10	5.43 ± 0.06	24.97 ± 0.02
	19	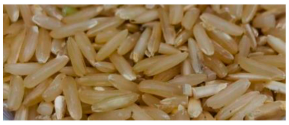	62.58 ± 0.04	6.02 ± 0.01	25.44 ± 0.02
	29	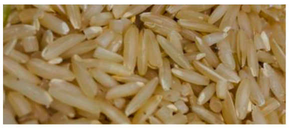	64.17 ± 0.12	5.48 ± 0.01	25.07 ± 0.05
	49	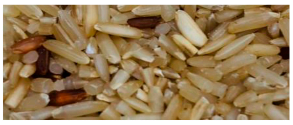	61.20 ± 0.04	5.78 ± 0.01	23.84 ± 0.01
	51	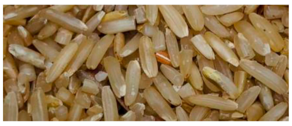	60.31 ± 0.58	5.81 ± 0.18	23.25 ± 0.19
	53	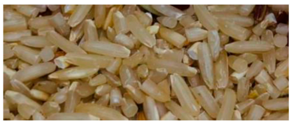	61.91 ± 0.19	5.13 ± 0.02	23.26 ± 0.03
	55	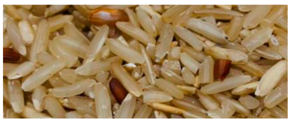	60.79 ± 0.25	6.08 ± 0.05	24.45 ± 0.09
	57	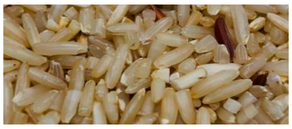	61.23 ± 0.07	5.78 ± 0.03	23.77 ± 0.05

Rice Varieties	Rice No.	Appearance	Color Analysis		
			L*	a*	b*
KDML105	T1	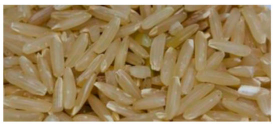	63.88 ± 0.05	4.86 ± 0.02	24.22 ± 0.05
	T2	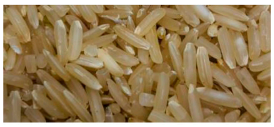	63.70 ± 0.01	5.11 ± 0.03	24.37 ± 0.07
	T4	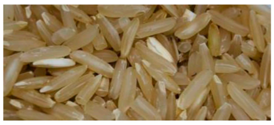	62.88 ± 0.21	5.10 ± 0.07	23.71 ± 0.08
	T6	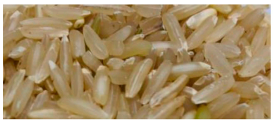	63.62 ± 0.13	4.83 ± 0.02	23.96 ± 0.11
	B2	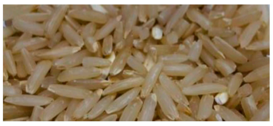	66.12 ± 0.23	4.56 ± 0.04	23.22 ± 0.03
	B11	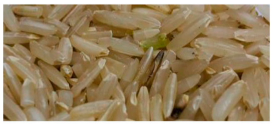	65.40 ± 0.10	4.19 ± 0.07	23.23 ± 0.03
	B14	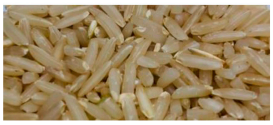	65.06 ± 0.03	4.65 ± 0.01	23.89 ± 0.03
	B18	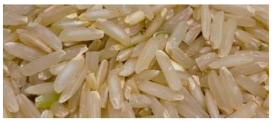	63.99 ± 0.02	4.80 ± 0.14	23.43 ± 0.03
RD15	31	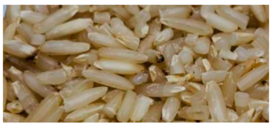	64.89 ± 0.03	4.43 ± 0.02	22.74 ± 0.02
	34	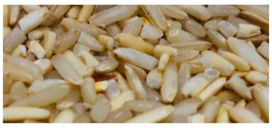	65.83 ± 0.14	4.94 ± 0.01	23.15 ± 0.10
	40	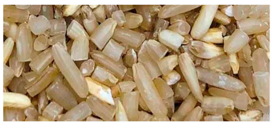	63.93 ± 0.31	4.91 ± 0.03	21.93 ± 0.04
	42	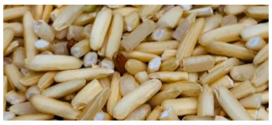	68.67 ± 0.36	5.15 ± 0.06	25.18 ± 0.11

Rice Varieties	Rice No.	Appearance	Color Analysis		
			L*	a*	b*
RD15	44	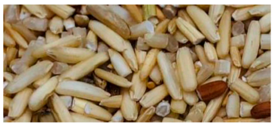	68.19 ± 0.13	5.75 ± 0.01	25.33 ± 0.07
	47	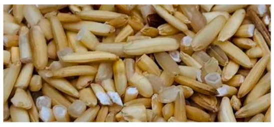	69.10 ± 0.08	5.47 ± 0.04	25.64 ± 0.14
	58	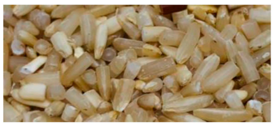	63.59 ± 0.03	5.22 ± 0.01	23.17 ± 0.06

Color analyses were determined using a ColorFlex EZ Spectrophotometer (Hunter Associates Laboratory, Reston, VA, USA) and expressed as CIELAB units (L* represents dark (0) to white (100) colors, a* represents green (-) to red (+) colors and b* represents blue (-) to yellow (+) colors). KDM105: Khao Dawk Mali 105; RD15: Rice Department 15.

Supplementary Table S2:

Table S2. Proximate compositions including energy and contents of protein, fat, carbohydrate, total dietary fiber, and ash of brown rice samples (per 100 g fresh weight).

Rice Varieties	Rice No.	Nutritional Composition of Brown Rice						
		Energy (kcal)	Moisture (g)	Protein (g)	Fat (g)	Carb (g)	TDF (g)	Ash (g)
KDML105	6	364.81 ± 0.47	10.78 ± 0.13	7.70 ± 0.00	2.73 ± 0.00	77.37 ± 0.13	1.54 ± 0.01	1.43 ± 0.00
	9	366.41 ± 1.04	10.45 ± 0.20	7.10 ± 0.02	2.87 ± 0.06	78.05 ± 0.14	1.59 ± 0.02	1.54 ± 0.02
	14	365.92 ± 0.05	10.58 ± 0.01	7.73 ± 0.03	2.82 ± 0.03	77.41 ± 0.09	2.54 ± 0.06	1.47 ± 0.01
	19	364.99 ± 0.41	10.81 ± 0.18	7.26 ± 0.05	2.91 ± 0.08	77.46 ± 0.33	2.53 ± 0.02	1.58 ± 0.02
	29	367.27 ± 0.94	10.67 ± 0.31	7.54 ± 0.06	3.21 ± 0.04	77.07 ± 0.25	3.02 ± 0.01	1.52 ± 0.03
	49	364.61 ± 0.10	11.17 ± 0.15	8.55 ± 0.01	3.09 ± 0.10	75.65 ± 0.24	2.94 ± 0.10	1.54 ± 0.00
	51	366.85 ± 0.13	11.12 ± 0.02	8.62 ± 0.04	3.45 ± 0.01	75.34 ± 0.06	2.93 ± 0.09	1.48 ± 0.01
	53	362.53 ± 0.23	11.26 ± 0.03	8.28 ± 0.04	2.71 ± 0.05	76.26 ± 0.09	2.98 ± 0.03	1.50 ± 0.03
	55	360.61 ± 0.31	11.36 ± 0.01	8.82 ± 0.09	2.57 ± 0.08	75.56 ± 0.00	2.94 ± 0.07	1.70 ± 0.01
	57	362.19 ± 1.38	11.21 ± 0.18	8.52 ± 0.02	2.89 ± 0.13	75.54 ± 0.09	3.19 ± 0.02	1.86 ± 0.02
	T1	362.65 ± 0.78	11.72 ± 0.00	7.12 ± 0.01	3.17 ± 0.15	76.42 ± 0.14	2.70 ± 0.01	1.58 ± 0.02
	T2	361.05 ± 0.04	11.98 ± 0.05	7.09 ± 0.01	2.93 ± 0.00	76.59 ± 0.01	2.63 ± 0.01	1.42 ± 0.04
	T4	361.19 ± 0.01	11.92 ± 0.03	6.81 ± 0.02	3.03 ± 0.05	76.69 ± 0.11	2.84 ± 0.01	1.57 ± 0.03
	T6	361.52 ± 0.68	11.80 ± 0.07	6.90 ± 0.03	2.90 ± 0.18	76.96 ± 0.20	2.56 ± 0.05	1.45 ± 0.02
	B2	366.46 ± 0.04	10.67 ± 0.04	6.92 ± 0.02	2.94 ± 0.04	78.08 ± 0.06	2.53 ± 0.01	1.39 ± 0.00
	B11	366.32 ± 0.46	10.59 ± 0.25	6.41 ± 0.06	2.94 ± 0.12	78.56 ± 0.45	2.50 ± 0.05	1.51 ± 0.02
	B14	364.04 ± 0.36	10.77 ± 0.08	7.11 ± 0.03	2.62 ± 0.03	78.02 ± 0.06	2.74 ± 0.03	1.49 ± 0.02
	B18	362.91 ± 1.90	11.22 ± 0.31	6.52 ± 0.02	2.69 ± 0.16	78.17 ± 0.10	2.94 ± 0.07	1.41 ± 0.03
RD15	31	361.62 ± 1.34	11.47 ± 0.03	6.79 ± 0.05	2.66 ± 0.26	77.64 ± 0.31	3.84 ± 0.00	1.45 ± 0.02
	34	363.46 ± 0.53	10.90 ± 0.14	7.17 ± 0.08	2.56 ± 0.01	77.95 ± 0.09	3.26 ± 0.14	1.43 ± 0.01
	40	364.36 ± 0.23	11.07 ± 0.09	7.03 ± 0.03	2.90 ± 0.03	77.54 ± 0.09	2.52 ± 0.03	1.47 ± 0.01
	42	366.52 ± 0.28	10.47 ± 0.04	9.06 ± 0.08	2.80 ± 0.09	76.29 ± 0.20	3.19 ± 0.09	1.40 ± 0.01
	44	366.29 ± 1.04	10.47 ± 0.22	8.93 ± 0.03	2.71 ± 0.02	76.56 ± 0.18	3.19 ± 0.14	1.35 ± 0.02
	47	365.63 ± 1.42	10.84 ± 0.41	8.95 ± 0.03	3.01 ± 0.04	75.69 ± 0.47	3.28 ± 0.03	1.52 ± 0.00
	58	362.43 ± 0.83	11.64 ± 0.22	7.42 ± 0.00	2.83 ± 0.01	76.82 ± 0.23	3.11 ± 0.14	1.30 ± 0.01

All data were represented as mean ± standard deviation (SD) of triplicate experiments (n = 3). KDM105: Khao Dawk Mali 105; RD15: Rice Department 15; Carb: carbohydrate; TDF: total dietary fiber.

Supplementary Table S3:

Table S3. Mineral and vitamin B3 contents of brown rice samples (per 100 g fresh weight)

Rice Varieties	Rice No.	Mineral Contents of Brown Rice (mg)						Vitamin B3 (mg)
		Ca	Mg	Na	K	Fe	Zn	
KDML105	6	8.56 ± 0.03	107.19 ± 0.53	12.88 ± 1.53	268.30 ± 3.32	0.88 ± 0.01	2.58 ± 0.01	2.65 ± 0.01
	9	6.29 ± 0.02	106.98 ± 1.98	12.71 ± 2.05	259.62 ± 1.68	0.66 ± 0.09	2.96 ± 0.35	2.77 ± 0.04
	14	7.32 ± 0.10	111.14 ± 2.53	18.63 ± 0.13	262.22 ± 0.30	0.95 ± 0.05	3.26 ± 0.23	2.60 ± 0.00
	19	7.01 ± 0.23	117.24 ± 0.61	17.33 ± 1.99	308.79 ± 3.60	1.07 ± 0.20	2.30 ± 0.07	3.40 ± 0.04
	29	6.86 ± 0.34	116.61 ± 0.98	10.13 ± 1.37	265.74 ± 6.85	0.86 ± 0.04	2.61 ± 0.04	2.95 ± 0.01
	49	8.43 ± 0.30	112.46 ± 2.05	22.87 ± 0.48	291.60 ± 4.74	1.48 ± 0.08	2.49 ± 0.06	3.80 ± 0.02
	51	8.71 ± 0.04	114.74 ± 2.69	27.68 ± 4.13	297.35 ± 9.09	1.45 ± 0.01	2.45 ± 0.06	2.66 ± 0.07
	53	8.46 ± 0.37	116.29 ± 0.70	21.05 ± 1.02	286.49 ± 3.19	1.19 ± 0.02	2.50 ± 0.02	2.38 ± 0.01
	55	10.66 ± 0.24	122.77 ± 0.14	29.89 ± 1.65	289.52 ± 3.46	2.13 ± 0.13	2.56 ± 0.03	3.70 ± 0.03
	57	12.03 ± 1.22	127.76 ± 1.26	23.11 ± 1.85	290.35 ± 2.14	1.83 ± 0.30	2.58 ± 0.01	3.61 ± 0.00
	T1	5.07 ± 0.01	115.89 ± 1.74	11.56 ± 2.34	209.44 ± 1.12	0.16 ± 0.02	1.83 ± 0.04	2.80 ± 0.02
	T2	5.33 ± 0.15	104.39 ± 3.43	10.52 ± 0.29	206.11 ± 4.55	0.30 ± 0.05	1.83 ± 0.03	2.07 ± 0.03
	T4	4.45 ± 0.42	87.02 ± 0.78	13.08 ± 0.25	207.30 ± 2.67	0.28 ± 0.00	1.81 ± 0.01	3.83 ± 0.02
	T6	5.54 ± 0.91	97.05 ± 16.52	8.95 ± 1.00	185.63 ± 14.10	0.43 ± 0.04	1.86 ± 0.07	3.87 ± 0.00
	B2	5.16 ± 0.08	97.98 ± 1.37	5.64 ± 0.54	188.66 ± 3.94	0.11 ± 0.04	2.25 ± 0.04	3.39 ± 0.03
	B11	5.51 ± 0.20	109.60 ± 6.57	5.09 ± 0.43	196.61 ± 7.43	0.41 ± 0.19	2.30 ± 0.08	3.60 ± 0.01
	B14	6.87 ± 0.10	104.47 ± 6.96	9.75 ± 1.42	192.61 ± 3.65	0.29 ± 0.02	2.30 ± 0.00	4.12 ± 0.05
	B18	6.41 ± 0.18	104.01 ± 1.69	10.24 ± 0.18	184.97 ± 3.26	0.08 ± 0.01	1.98 ± 0.06	4.11 ± 0.00
RD15	31	8.08 ± 0.31	114.19 ± 1.06	10.63 ± 0.21	249.14 ± 0.83	0.70 ± 0.12	2.33 ± 0.01	2.90 ± 0.06
	34	11.91 ± 0.57	111.05 ± 0.92	26.65 ± 0.02	220.57 ± 0.31	1.14 ± 0.07	2.08 ± 0.02	2.54 ± 0.11
	40	11.50 ± 0.75	109.59 ± 0.79	23.52 ± 1.08	233.99 ± 4.23	0.93 ± 0.01	1.90 ± 0.00	3.66 ± 0.29
	42	9.29 ± 0.47	86.64 ± 1.51	32.67 ± 2.92	220.95 ± 2.92	1.30 ± 0.21	2.19 ± 0.03	2.40 ± 0.05
	44	8.41 ± 0.70	85.10 ± 6.02	31.69 ± 1.27	223.06 ± 5.82	1.12 ± 0.14	2.16 ± 0.06	3.30 ± 0.02
	47	10.55 ± 0.19	106.33 ± 1.23	30.62 ± 0.62	230.99 ± 0.35	1.09 ± 0.14	2.23 ± 0.02	3.98 ± 0.05
	58	9.17 ± 0.23	95.06 ± 3.71	22.99 ± 0.07	186.77 ± 3.65	1.35 ± 0.06	1.92 ± 0.03	4.32 ± 0.01

All data were represented as mean ± standard deviation (SD) of triplicate experiments (n = 3). KDM105: Khao Dawk Mali 105; RD15: Rice Department 15.

Supplementary Table S4:

Table S4. Proximate compositions of brown rice samples (KDML105 and RD15 varieties) compared with data from the Thai Food Composition Database (FCD) and the U.S. Department of Agriculture (USDA) FDC Databases (per 100 g dry weight).

Samples	KDML105 variety (min-max)	RD15 variety (min-max)	Jasmine variety (Thai FCD [a])	Unknown varieties (Thai FCD [a])	Unknown varieties (USDA FDC [b])
Energy (kcal)	407.89-412.74	407.92-410.15	411.2	409.3	412.4
Protein (g)	7.17-9.95	7.66-10.11	8.16	8.48	8.55
Fat (g)	2.89-3.88	2.87-3.38	3.28	3.07	3.63
Carbohydrate (g)	84.77-88.04	84.89-87.69	87.24	86.94	86.39
Dietary fiber (g)	1.73-3.59	2.83-4.34	3.89	4.44	4.08
Ash (g)	1.56-2.09	1.47-1.70	1.33	1.51	1.37
Calcium (mg)	5.05-13.55	9.13-13.36	10.01	15.93	10.20
Sodium (mg)	5.69-33.71	12.01-36.48	32.26	12.51	5.67
Potassium (mg)	208.34-346.20	211.36-281.41	193.55	311.72	283.45
Magnesium (mg)	98.79-143.88	95.06-128.98	NA	67.12	131.52
Iron (mg)	0.09-2.40	0.79-1.52	1.31	1.15	1.46
Zinc (mg)	2.05-3.65	2.14-2.63	NA	0.57	2.41
Vitamin B3 (mg)	2.35-4.63	2.68-4.89	5.38	6.30	7.36

[a] Thai Food Composition Database: Brown rice, Jasmine variety, raw (Food code A1), Brown rice, different unknown varieties, raw (Food code A9), calculated to dry weight basis using moisture content of 10.1 and 12.1%, respectively.

[b] U.S. Department of Agriculture (USDA), Food Data Central (FDC) Database: Long-grain brown rice, unknown varieties, raw (FDC ID 169703), calculated to dry weight basis using moisture content of 11.8%.

NA = data not available
